# Formation of Cysteine Adducts with Chlorogenic Acid in Coffee Beans

**DOI:** 10.3390/foods13111660

**Published:** 2024-05-25

**Authors:** Sorel Tchewonpi Sagu, Nina Ulbrich, Johanna Rebekka Morche, Kapil Nichani, Haydar Özpinar, Steffen Schwarz, Andrea Henze, Sascha Rohn, Harshadrai M. Rawel

**Affiliations:** 1 Institute of Agricultural and Nutritional Sciences, Martin Luther University Halle-Wittenberg, Von-Danckelmann-Platz 2, 06120 Halle (Saale), Germany; sorel.sagu@landw.uni-halle.de (S.T.S.); andrea.henze@landw.uni-halle.de (A.H.); 2 Institute of Nutritional Science, University of Potsdam, Arthur-Scheunert-Allee 114-116, 14558 Nuthetal, Germany; nina.ulbrich@tu-berlin.de (N.U.); johanna.rebekka.morche@uni-potsdam.de (J.R.M.); kapil.nichani@uni-potsdam.de (K.N.); 3 Institute of Food Technology and Food Chemistry, Technische Universität Berlin, Gustav-Meyer-Allee 25, 13355 Berlin, Germany; rohn@tu-berlin.de; 4 Department of Nutrition and Dietetics, Faculty of Health Sciences, İstanbul Aydın Üniversitesi, Mah. İnönü Cad. No: 38 Sefaköy, 34295 İstanbul, Turkey; haydarozpinar@aydin.edu.tr; 5 Coffee Consulate, Hans-Thoma-Strasse 20, 68163 Mannheim, Germany; schwarz@coffee-consulate.com

**Keywords:** coffee beans, caffeoylquinic acids, oxidation, isomerization, cysteine adducts, stability and identification

## Abstract

The post-harvest processing of coffee beans leads to a wide range of reactions involving proteins. The formation of crosslinks between proteins and phenolic compounds present in high concentrations of coffee beans represents one of the most challenging and still not fully characterized reactions. The aim of this work was to assess the presence of products from such reactions in coffee samples, focusing on the adducts between cysteine and chlorogenic acids (CQAs). For this purpose, 19 green and 15 roasted coffee samples of the *Coffea arabica*, *Coffea canephora*, and *Coffea liberica* varieties were selected for this study and basically characterized. Then, targeted liquid chromatography mass spectrometry (LC-MS/MS) methods were developed to assess the formation of adducts between CQA and cysteine, glutathione, and N-acetylcysteine as the amino acid and peptide models, and quantified such adducts in coffee samples. The results of the characterization showed a heterogeneous distribution of the protein content (8.7–14.6%), caffeine (0.57–2.62 g/100 g), and antioxidant capacity (2–4.5 g ascorbic acid/100 g) in *Arabica*, *Canephora*, and *Liberica* samples. Glutamic acid, arginine, and proline were found to be the major amino acids, while 5-CQA (38–76%), 3-CQA (4–13%), and 4-CQA (4–13%) were the most abundant CQA derivatives of all coffee varieties. The model experiments for adduct formation demonstrated that cysteine binds to CQA via thiol groups and 5-CQA initially isomerizes to 3- and 4-CQA, depending on the conditions, allowing cysteine to bind to two different sites on 3-, 4- or 5-CQA molecules, thus, forming six different Cys-CQA adducts with *m*/*z* 476. The reaction was more favored at pH 9, and the adducts proved to be stable up to 90 °C for 10 min and up to 28 days at room temperature. The relative quantification of adducts showed peak area values ranging from 1100 to 3000 in green coffee bean samples, while no adducts were detected in roasted coffee beans. Overall, this work was the first attempt to demonstrate the presence of Cys-CQA adducts in coffee beans and paves the way for further investigations of such adduct formation at the protein level.

## 1. Introduction

Coffee cultivation, which originated in Lower Guinea with the first cultivations dating back 1500 years, has considerably evolved over time, resulting today in an estimated number of 134 coffee species of the genus *Coffea* L. [1]. Nowadays, coffee is the world’s second most-traded commodity after crude oil [2]. Commercially relevant species are *Coffea arabica* and *Coffea canephora*—which account for more than 95% of the global production—whereas, in the last few years, *Coffea liberica* has also gained some attention [3]. However, the latter is still of very low economic importance and represents less than 1% of global production [4]. In the season 2021/2022, the global coffee harvest volume amounted to over 10 billion kilos [5].

Mainly consumed as a beverage, coffee is highly appreciated not only for its taste but also for its potential health benefits. It has been shown that nutritional and functional properties, as well as the chemical components of coffee beans, play an important role [6,7,8]. A recent compilation of the available information focused in this context on the functional properties of coffee, coffee beans, and by-products in terms of the associated potential health benefits [9]. Among bioactive constituents, caffeine, micronutrients, diterpenes, and phenolic compounds, including hydroxycinnamic acid derivatives known as antioxidants, are the most relevant [10,11,12]. Phenolic acids belong to the group of antioxidants, which are rarely found in isolated form but rather as cinnamoylquinic acids after esterification with quinic acid. In a narrower sense, these esters of caffeic acid with quinic acid are generally termed chlorogenic acids. In the literature, this term often finds further extension, and the group of chlorogenic acids may include esters of quinic acid with other hydroxycinnamic acids, such as ferulic and p-coumaric acid. Therefore, commonly found chlorogenic acids in coffee include caffeoylquinic acids (CQA), existing in three isomeric forms (3-CQA, 4-CQA, and 5-CQA), the derivatives of dicaffeoylquinic acids (diCQA) with three respective isomers (3,4-diCQA, 3,5-diCQA, and 4,5-diCQA) and feruloylquinic acids with three isomers 3-FQA, 4-FQA, and 5-FQA [13]. Recent studies on the composition of phenolic compounds in coffee revealed the presence of over 50 different hydroxycinnamic acid derivatives [14,15]. During the roasting process, CQAs are progressively degraded and transformed, releasing a series of reactive products [16,17]. Despite such deterioration, Clifford [11] showed that, depending on roasting conditions, substantial amounts of chlorogenic acids remain in roasted coffee beans, ensuring a daily intake that can vary from a few milligrams to almost one gram, only with coffee consumption alone.

Phenolic compounds are quite reactive. Their chemical reactivity, especially towards proteins and free amino acids, derives from their ability to easily oxidize enzymatically or chemically [18,19]. The occurrence of a catechol moiety, i.e., the two adjacent (ortho) aromatic hydroxyl groups, and the subsequent enzymatic oxidation of these to reactive and redox-active *o*-quinones, appears to be a pre-requisite chemical–structural condition, generating electrophilic species that are capable of undergoing a nucleophilic addition to proteins [12]. The complexity of following up this type of reaction in coffee-based food matrixes arises from the fact that the major phenolic compound present in coffee cherries and beans, 5-CQA, is liable to isomerization (Table S1, Supplementary Data) and oxidation, thus producing a series of reaction products itself, which have hardly been characterized [20]. However, these, in turn, react in different ways with individual amino acids or protein side chains, further increasing the diversity of reaction products.

The reaction of phenolic compounds with free amino acids can proceed to form different colored adducts, depending on the pH conditions documented by Bongartz et al. [21]. HPLC, in combination with tandem mass spectrometry (HPLC-MS/MS), was used to characterize these adducts, where six different forms were found to be dominant and allocated to the green trihydroxy benzacridine derivatives as postulated and structurally elucidated in previous studies [22,23]. As the quinone form of CQA tends to form a dimer, it reacts with a primary amino group, as indicated by Yabuta et al. [18], or in the case of proteins with the ε-amino group of the protein side chains of lysine [19,20]. Due to its highly nucleophilic character, cysteine reacts preferably with CQA monomers [21,23]. In this context, a recent study reported on the synthesis, purification, and characterization of a chlorogenic acid-cysteine (CQA-Cys) adduct formation with a characteristic molecular species of *m*/*z* 474.1 [24]. Thus, the development of mass spectrometric methods has recently provided a more detailed biochemical characterization of the interactions of CQA and amino acids [21,23].

Given that the diversity of the different hydroxycinnamic acid derivatives present in coffee beans complicates our understanding of the reactions taking place, providing a wide range of reaction products, the primary objective of this work was to monitor and quantify the formation of CQA adducts that may occur in coffee beans. To this end, coffee beans of different genotypes, representing different amounts and compositions of CQA, were subjected to different post-harvest treatments. The methodology applied consisted initially of characterizing the coffee samples in terms of their amino acid and chlorogenic acid composition. Subsequently, targeted LC-MS/MS methods were developed to characterize the adducts of chlorogenic acids with cysteine, N-acetylcysteine, and glutathione in model systems. These adducts were finally analyzed and quantified in green and roasted coffee bean extracts.

## 2. Materials and Methods

### 2.1. Coffee Samples and Chemicals

#### 2.1.1. Coffee Samples

A total of 19 green coffee samples and 15 roasted coffee samples of the species *Coffea arabica*, *Coffea canephora*, and *Coffea liberica* were used for analysis. Table S2 (Supplementary Data) presents the description of the analyzed coffee samples. The coffee varieties Old Paradenia (*Canephora*), S795 (*Arabica*), and a *Liberica*, which originated from the two fields Kerkiecoondah (KC) and Balehonnur (BHR) from India, were kindly provided by the company Coffee Consulate (Mannheim, Germany). These samples were fermented with known microorganisms (*Candida krusei* and *Opus palatina*), and the coffee beans were roasted at 202 °C for approx. 14 min. Twelve commercially available green coffee beans (*Coffea arabica*) from different countries were purchased from Coffeewell GmbH (Mettmann, Germany). Further green and roasted beans from the species *C. arabica* and *C. canephora* were kindly provided by the Istanbul Aydin University (Istanbul, Turkey). All coffee samples were gently ground with dry ice and fractionated using an ultra-centrifugal mill (Retsch GmbH, Haan, Germany) to obtain flours with a particle size of <0.2 μm.

#### 2.1.2. Chemicals

Caffeine, 5-CQA, and L-glutathione were obtained from Carl Roth GmbH & Co. KG (Karlsruhe, Germany). Amino acids of standard solution, N-acetyl-L-cysteine and L-cysteine, were purchased from Merck KGaA (Darmstadt, Germany). The solvents used for mass spectrometry analysis were of LC-MS grade, and all the other chemicals were of analytical grade.

### 2.2. Preliminary Analysis

The raw protein content was determined using the Kjeldahl method, applying a factor of 5.5 to convert the total nitrogen in the protein content. The dry matter was determined after drying the samples at 105 °C to a constant weight.

#### 2.2.1. Determination of the Antioxidative Capacity of the Coffee Bean Extracts

Analysis was performed to encompass free and bound CQA. For the determination of antioxidant capacity, the FRAP assay with ascorbic acid as a reference substance was used. The method was adapted, as described in a recent study [2]. The measurement was performed using a plate reader (Power Wave XS2, BioTek Instruments GmbH, Bad Friedrichshall, Germany) and the associated data acquisition and analysis software Agilent Biotek Gen5 (Vers.: 3.x, Latest Vers. 3.15.15, 2023, Agilent Technologies Deutschland GmbH, Waldbronn, Germany).

#### 2.2.2. Free Amino Acids, Caffeine and Chlorogenic Acid Determination

##### Free Amino Acids

Free amino acids were determined by mixing 10 mg of coffee samples with 1 mL of 50% acetonitrile in water for 10 min at room temperature and under shaking conditions. The samples were centrifuged (4 °C, 10 min, 10,000× *g*) and the supernatants were collected. Thereafter, 100 μL of the extracts were mixed with 880 μL of 50% acetonitrile and 20 μL of 1 mM BOC-tyrosine (BOC-Tyr) in 0.1% formic acid. Prior to the analysis, the mixture was furthermore diluted with a solution of 50% acetonitrile in a ratio of 1:10 (*v*/*v*). BOC-tyrosine was used as an internal standard to normalize the data. Quantification was performed by carrying out an external calibration with a standard solution containing all 20 proteinogenic amino acids in the concentration range of 0.1 to 6 µM of each amino acid. The analytic information related to the conditions of the amino acids analysis is given in Table S3 (Supplementary Data).

##### Analysis of Caffeine and CQA Derivatives

To determine the content of caffeine, CQA, and the distribution of CQA isomers, 10 mg of each coffee sample was mixed twice with 1 mL of 80% methanol in water containing 1% formic acid (FA) (80:20 *v*/*v*). The extraction was performed under shaking at room temperature for 30 min. After centrifugation at 9300× *g* for 10 min, the supernatants were collected and pooled in a 2 mL reaction tube and stored at −20 °C. Prior to the analysis of the caffeine and CQA contents, extracts were additionally diluted 1:10 with 80% methanol in water containing 1% FA (80:19:1 *v*/*v*/*v*). Calibration was performed using a dilution series of 5CQA and caffeine in 80% methanol in water containing 1% FA (80:19:1 *v*/*v*/*v*) within the range of 5–40 μg/mL for 5CQA and 5–30 μg/mL for caffeine. A method combining mass spectrometry detection with a UV-Vis detection was developed for quantification. The method was extended to include more precise information on individual CQA based on recently published studies [2]. The UV detection of analytes was performed at 280 nm (caffeine) and 325 nm (CQA), and the results were expressed as g/100 g. To carry out these analyses, an Agilent Infinity 1260 HPLC system was used with an UV detector in combination with an Agilent G6470A Series Triple Quad mass spectrometer (Agilent Technologies Deutschland GmbH, Waldbronn, Germany). The detailed HPLC (including the column and eluents used) and MS conditions (including the transition lists) for the analysis of the CQA and caffeine are given in Table S4 (Supplementary Data).

### 2.3. Analysis of CQA-Adducts

The reaction model was performed by mixing 100 mM of a cysteine stock solution with 30 mM CQA at a ratio of 1:1 (*v*/*v*), and the blank solutions were prepared by mixing the stock solutions with distilled water (ratio 1:1, *v*/*v*). After adjusting the pH of the solutions to the desired pH value with sodium hydroxide, they were then incubated for 30 min at room temperature. The pH was readjusted, if necessary, and the samples were incubated for 20 h at room temperature. Finally, 500 μL of 1% formic acid was added to stop the reaction. After centrifugation at 10,000× *g* for 5 min, the supernatants were collected and diluted with distilled water (ratio 1:10, *v*/*v*) prior to the analysis. For the analysis of CQA adducts in coffee beans, samples were processed, as described in Section Analysis of Caffeine and CQA Derivatives, and the original samples were diluted 1:10 with distilled water prior to the analysis.

A targeted LC-MS/MS method was specifically developed in order to analyze the adducts. The workflow applied, as well as the detailed HPLC information (including the column and eluents used) and MS conditions (including the transition lists), are given in Table S5 (Supplementary Data).

### 2.4. Effect of the pH Value and Concentration

These experiments were undertaken in order to determine the most suitable conditions for adduct formation. For this purpose, different pH values (2, 5, 6, 7, 9, 10, 11, and 12) as well as different molar ratios of CQA and cysteine standard solutions (0.1, 0.2, 0.3, 0.4, 0.5, 0.6, and 1) were investigated.

### 2.5. Stability of the Cys-CQA-Adducts

The stability of the adducts was investigated following three different procedures: (I) after the reaction was stopped, the samples were heated at different temperatures (37, 60, and 90 °C) for 30 min before measurements; (II) the solutions were treated with microwaves (Discover SP-D 80, CEM GmbH, Kamp-Lintfort, Germany) at 37, 60, and 90 °C (ramp time of 5 min and hold time of 10 min at a pressure of 97 kPa and 300 W); and (III) the solutions were stored either at room temperature or at −20 °C for two months and the samples were measured at several time points.

### 2.6. Determination of N-Acetylcysteine Modifications as a Function of pH Value

The difference between N-acetylcysteine (NAC) and cysteine is that the amino group is blocked through acetylation, and thus, CQA can only react with the thiol group. These experiments were undertaken in order to clarify the binding site of cysteine to chlorogenic acid. For this purpose, 100 mM of the NAC stock solution and a 30 mM CQA stock solution were mixed at a ratio of 1:1 (*v*/*v*). The blanks were prepared by mixing each individual stock solution with distilled water at a ratio of 1:1 (*v*/*v*). All solutions were then adjusted to the desired pH value with sodium hydroxide and incubated for 30 min at room temperature. After readjusting the pH, if necessary, the solutions were further incubated for 20 h at room temperature. The reaction was stopped by adding 1 mL of 1% formic acid; the samples were centrifuged at 10,000× *g* for 5 min, and the supernatants were collected and pooled. Prior to the analysis, the samples were diluted 1:10 with distilled water. The detailed HPLC (including column and eluents used) and MS conditions (including the transition lists) for the analysis of NAC-CQA adducts are given in Table S6 (Supplementary Data).

### 2.7. Determination of Glutathione-CQA Modifications as a Function of pH Value

Glutathione (GSH) is a small peptide with a molar mass of 307.33 g/mol. In addition, glutathione is ubiquitously present in animal and plant cells and should, therefore, also be detected in coffee beans. The aim of this series of investigations was to observe how the adduct formation of chlorogenic acid with glutathione behaves. For this purpose, the sample preparation protocol was the same as previously described in Section 2.3, and pH values of 5, 7, 9, and 12 were tested with regard to adduct formation. The detailed HPLC-MS/MS conditions are given in Table S7 (Supplementary Data).

### 2.8. Data Analysis

All experiments were performed in triplicate, and data are expressed as the mean ± standard deviation. The data analysis was performed with the Skyline software, version 23.1 [25]. The results were analyzed with GraphPad Prism 8^®^ (GraphPad Software, San Diego, CA, USA) while applying two-way ANOVA and Tukey’s test, using a statistical significance set at *p* < 0.05.

## 3. Results and Discussion

### 3.1. Preliminary Investigations

#### 3.1.1. Dry Matter, Antioxidant Capacity, Protein and Caffeine Contents

The aim of the basic analysis was to initially characterize the selected coffee samples. To this end, a range of analyses were carried out, including the determination of the dry mass, the antioxidant capacity as well as the raw protein, caffeine, amino acid, and CQA contents. Table 1 presents a summary of the results obtained.

The roasted coffee samples contained a higher proportion of dry matter compared to green coffee beans, and the contents were comparable between the varieties. A moisture content of 7–13% for the chemical composition of raw coffee beans of the *Arabica* and *Canephora* varieties has been reported, leading to dry matter in the range of 87–93% [26]. These values were confirmed for green coffee beans in general (88.9–93.2%; Figure S1). Dry matter of 95.6–98.7% (Table 1) was found in roasted coffee beans, whereby, again, no significant differences between the varieties could be identified. It was shown that after roasting, coffee beans only contained a residual moisture content of 1.5–3.5%, corresponding to a dry matter content of 96.5–98.5% [26].

When performing Kjeldahl analysis, the values of other nitrogenous substances in coffee, such as caffeine, trigonelline, and free amino acids, are generally included (Table 1). Deducting the non-protein nitrogen content of 1.2–3.8%, protein content values of 12–14.6%, 8.7–11.3%, and 8.9–11.5% were recorded for *Canephora*, *Arabica*, and *Liberica* green coffee beans, respectively (Figure S2, Supplementary Data). The raw protein content of the *Canephora* and *Arabica* samples increased after roasting, while the protein content of the *Liberica* samples remained constant. The observed differences may be explained by various factors, including species, geographical origin, post-harvest processing methods, the degree of ripeness of the coffee cherries, and processing techniques; storage-specific studies to encompass these aspects are still lacking.

It was observed that the values of the antioxidant capacity ranged from 2 to 4.5 g ascorbic acid/100 g (Figure S3). *Canephora* green coffee beans showed higher values of antioxidant capacity compared to the *Arabica* samples. The roasted samples showed higher values of antioxidant capacity in all varieties compared to their corresponding green coffee beans. This increase could be due to the fact that other antioxidants, such as melanoidins, are clearly produced during the roasting [27].

When analyzing samples by LC-MS/MS, higher caffeine contents were found for all samples compared to the UV analysis at 280 nm (Figure S4). An exemplary UV chromatogram (280 nm) and a total ion chromatogram (TIC) for caffeine are provided in Figure S5. With up to 1.79 g of caffeine per 100 g of green coffee and up to 2.62 g of caffeine per 100 g of roasted coffee, *Canephora* samples showed high caffeine content with both methods. *Liberica* variety tends to have the lowest caffeine content in green coffee beans (0.54–0.80 g/100 g DM) as well as in roasted coffee (0.64–0.86 g/100 g DM). *Arabica* varieties, on the other hand, yielded values of 0.57–1.21 and 0.63–1.45 g/100 g DM in green and roasted coffee samples, respectively.

#### 3.1.2. Content of Free Amino Acids

**Figure 1:**
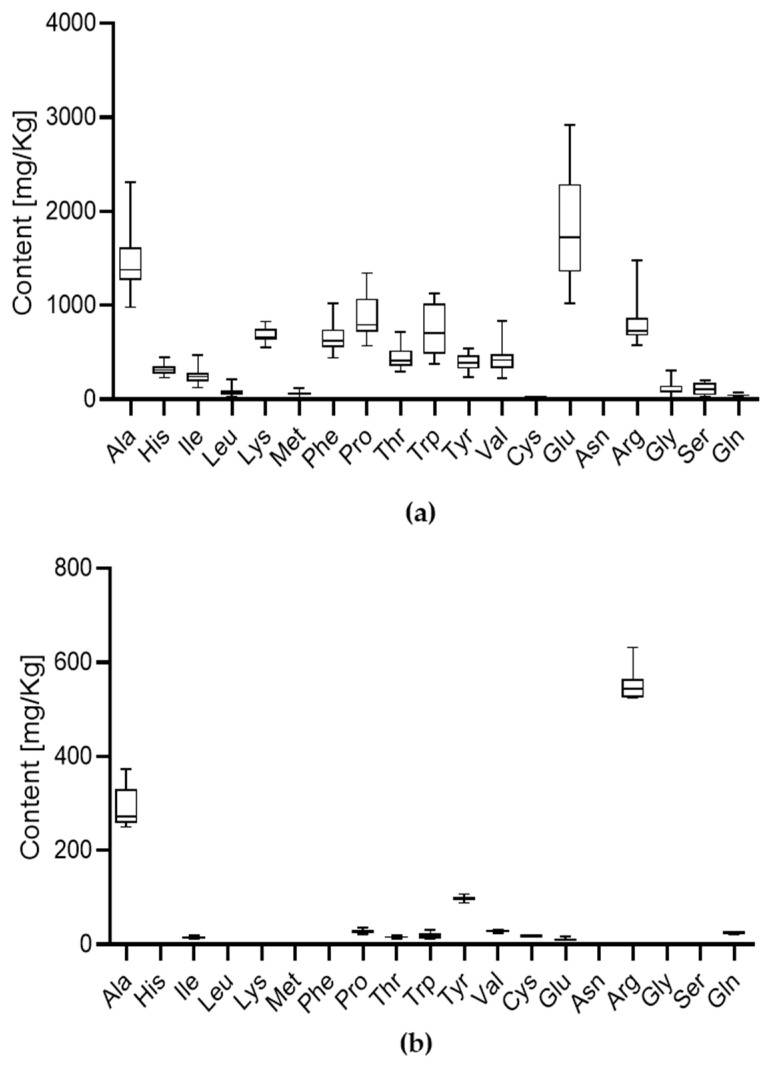
Free amino acids in (**a**) green and (**b**) roasted coffee based on dry matter.

Free amino acids were analyzed in all the selected green and roasted coffee beans, and the results are given in Figure 1. Glutamic acid (1022–2921 mg/kg DM) and alanine (982–2312 mg/kg DM) showed high contents in green coffee (Figure 1a). Arginine and proline residues presented maximum values of 1481 and 1343 mg/kg DM, respectively. Leucine (24–214 mg/kg DM), methionine (20–117 mg/kg DM), asparagine (0.3–2 mg/kg DM), and cysteine (0–23 mg/kg DM) had among the lowest values. In roasted coffee beans, the levels of free amino acids were further decreased. Alanine, arginine, and tyrosine values ranged from 250 to 373, 523 to 631, and 89 to 107 mg/kg DM, respectively. Leucine, lysine, methionine, phenylalanine, histidine, and asparagine were not detected in roasted samples, while the other amino acids were only present in small quantities (up to 36 mg/kg DM). This was due to the formation of browning products in the Maillard reaction and a general degradation during roasting [27,28]. Li et al. (2022) were able to demonstrate, among others, that acrylamide formation from the amino acid lysine can also occur [29]. Arnold et al. [30] identified aspartic acid and glutamic acid as the most abundant free amino acids, but proline, alanine, and histidine were also detected in high quantities. Free amino acids were reported only in traces in roasted coffee [26].

The results obtained are partially consistent with the literature. Glutamic acid is the most abundant free amino acid of coffee beans, and this could also be confirmed for alanine and proline. It should be mentioned that only low levels of cysteine in their unbound form were detected in some samples.

#### 3.1.3. Content of Chlorogenic Acid Derivatives

**Figure 2:**
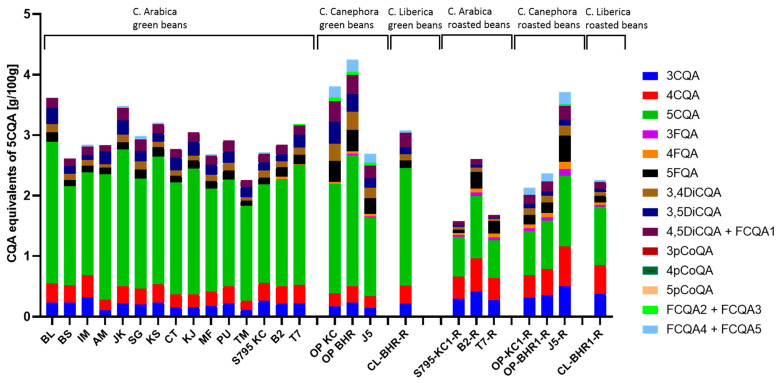
Determination of the CQA under UV-Vis conditions; abbreviations: CQA—caffeoylquinic acid, DiCQA—di-caffeoylquinic acid, FCQA—feruoylcaffeoylquinic acid, FQA—feruoylquinic acid, and pCoQA—p-coumaroylquinic acid. The amount is given as 5-CQA equivalents in g per 100 g of dry matter.

Green coffee also contains a variety of phenolic compounds, where the main components are chlorogenic acids, among which the CQA, especially 5-CQA is with the highest concentration with lower amounts of feruloylquinic acids (FQA) and dicaffeoylquinic acids (Di-CQA), as illustrated in Figure 2. Typical chromatograms are presented in Figure S5. When analyzing samples by MS, higher 5-CQA contents were found for all samples compared to the UV analysis at 325 nm (Figure S6).

**Figure 3:**
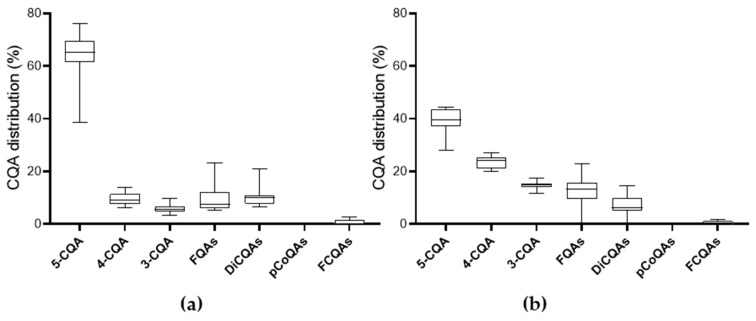
Chlorogenic acid distribution in the coffee samples during MRM measurement—(**a**) green; (**b**) roasted coffee beans. abbreviations: CQA—caffeoylquinic acid, DiCQA—di-caffeoylquinic acid, FCQA—feruoylcaffeoylquinic acid, FQA—feruoylquinic acid, and pCoQA—p-coumaroylquinic acid.

According to the literature, CQA accounts for 6–12% of the dry matter of coffee beans [2,26]. In green *Arabica* beans, 4.0–8.4% based on dry weight were reported [31,32]. Although the ratios are correct, the CQA content determined in the present study was lower than that reported in the literature. Contrary to this, the CQA distribution (Figure 3a) found in the present study is in line with other studies. In their review, Farah & Donangelo (2006) summarized the results of various studies and came to a comparable conclusion regarding the content of the nine main CQA isomers in green coffee beans [31].

In their work, Clifford et al. (2003) described a total of 18 different CQAs (Table S1) [33]. Of these, 16 were detected by HPLC combined with mass spectrometry (MS) in the samples of the present study, but only 14 could be quantified. It was not possible to identify all six FCQAs (as no standards were available). Only five FCQAs were found by the MS/MS (MRM) method. Furthermore, an exact assignment of the FCQA was not possible due to the fragmentations followed by the MRM method, which is why the designation of FCQA 1–5 used here does not correspond to the assignment described by Clifford et al. but only to the order of elution [33].

5-CQA was the most abundant CQA and accounted for approximately 56–62% of the total CQA found in green coffee beans. 3-CQA and 4-CQA were each responsible for up to 10%. Di-CQA isomers accounted for around 15–20% of the total CQA in green coffee beans, and FQA isomers made up 5–13% of the total CQA. The other CQAs together made up the remaining percentage (Figure 3a).

The CQA composition differed slightly depending on the variety. For example, the occurrence of FCQA was characteristic of the *Canephora* coffee variety, whereas the *Arabica* variety was reported to contain no FCQA at all [11]. This could not be confirmed for green coffee in the present study, as some *Arabica* samples, as well as the *Liberica* samples, showed a low proportion of FCQA. For roasted coffee, on the other hand, it could be confirmed that only *Canephora* still contained FCQA.

Roasting shifted the distribution of CQA, and the content was found to be similar among the coffee varieties (Figure 3b). The most abundant CQA was 5-CQA (28–43%), while amounts of 3-CQA and 4-CQA were found to be around 11–17% and 20–26%, respectively. Di-CQA isomers were present in much lower proportions than their CQA monomers. 5-FQA was the most common FQA isomer (4–13%), and FCQA and pCoQA were only found in small quantities. Kwon et al. (2015) noticed a loss of CQA in the roasted coffee samples [34], which was confirmed in the present study. However, as CQAs are precursors for further complex redox-based reactions, this decline in their concentration was predictable. Through autoxidation at high temperatures (roasting), phenolic compounds and especially hydroxycinnamic acids, which are both strong antioxidants, are oxidized to quinones, which can then react to form browning products or bind to the reactive side chains of the proteins [12]. Clifford (2000) noted that the higher the degree of roasting, the more the CQA content of the coffee beans decreased [11]. Awwad et al. (2021) came to a similar conclusion [16]. Farah et al. (2005) also observed that the 5-CQA content decreased when the green beans were lightly roasted, while the content of the isomers 3-CQA and 4-CQA increased at the same time [35]. The roasted coffee beans tested in the present study, for which green coffee beans of the same origin and processing were also available as reference samples, were roasted for 13–14 min at a temperature of approx. 202 °C. For these samples, an approx. 50% loss of total CQAs was observed, which reflects the results of light roasting described by Awwad et al. (2021) [16].

### 3.2. Formation of Cysteine CQA Adducts

**Figure 4:**
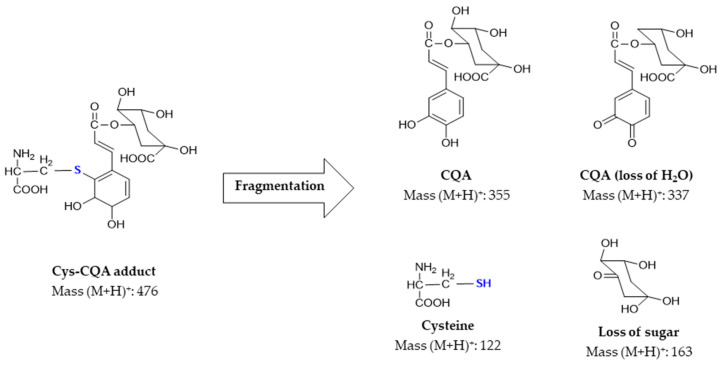
Proposed MS/MS fragmentation pattern of the *m*/*z* 476 in positive mode for the Cys-CQA adduct, with product masses tracked in the optimized method. CQA—caffeoylquinic acid; Cys—cysteine.

So far, it has been shown that among the free amino acids, very low amounts of cysteine (0–23 mg/kg DM) were determined, and these were found only in a few selected coffee samples. On the other hand, large amounts of 5-CQA were found in all samples, thus opening up the perspective that these two components would interact to form covalent bound adducts. To investigate adduct formation, standard solutions of 5-CQA and cysteine were initially used to assess the formation of the adducts. Figure S7 presents the chromatograms for 5-CQA, cysteine, and the product of the reaction obtained. In the mass spectrum of 5-CQA and cysteine, several new peaks were observed not belonging to pure CQA or cysteine. The following optimization method led to the characterization of six prominent peaks with characteristic precursor ions of *m*/*z* 476 and *m*/*z* 951 with similarities in fragmentation patterns (Table S5, Supplementary Data). An exemplary extracted ion chromatogram for *m*/*z* 476 and the corresponding product ions are provided in Figure S7B,C). Figure 4 shows the proposed typical fragmentation pattern for the Cys-CQA adducts with *m*/*z* 476.

Among the breakdown products, both cysteine (*m*/*z* 122) and CQA (*m*/*z* 355) were identified in the positive mode. However, CQA was also successfully monitored in the negative mode (*m*/*z* 353). The fragment of *m*/*z* 162 corresponding to quinic acid was formed by cleavage of caffeic acid. The fragment with *m*/*z* 336 resulted from the loss of one molecule of water from the CQA during fragmentation. Additional product ions were obtained from the fragmentation of the adduct, but it was not possible to identify the corresponding compounds or to correlate these fragments with their tentative chemical structures. The molecules shown here are only the most frequently occurring ones under these MS/MS settings and those recorded in the MRM method for clear identification. The second identified precursor with *m*/*z* 951 involves two moles of CQA with two moles of cysteine. This precursor yielded six different chromatographic peaks corresponding to the retention times of 7.5, 8.1, 11.2, 11.6, 11.9, and 12.9 min. The fragmentation of this precursor revealed that it was most likely a polymerization product in which further oxidation may have led to the adduct formation of two molecules of *m*/*z* 476. As the retention times are exactly the same as those of the monomer *m*/*z* 476, ion clustering would also explain the observed precursor *m*/*z* 951.

**Figure 5:**
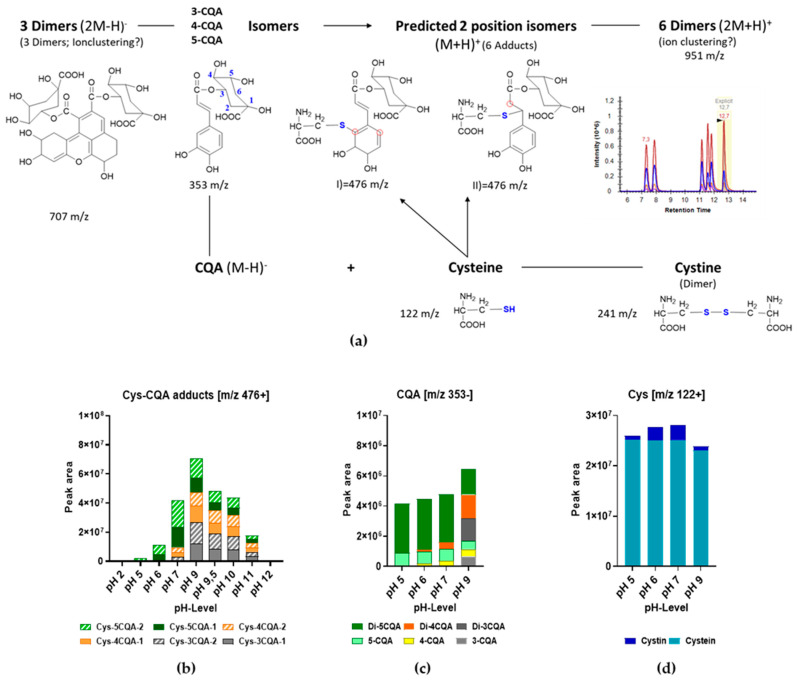
Reaction of CQA with cysteine as affected by pH in a 1:1 mixture (*v*/*v*) of 30 mM 5-CQA and 100 mM cysteine incubated for 20 h. (**a**) Major reaction products were monitored, and the red rings indicate possible reaction sites; (**b**) effect of pH on adduct formation; (**c**) iso- and dimerization observed in the blank CQA solutions; (**d**) dimerization in the blank cysteine solutions. Abbreviations: CQA—caffeoylquinic acid; Cys—cysteine. Digits 1 and 2 in (**b**) correspond to the two possible position isomers.

Under alkaline pH conditions, 5-CQA can isomerize into 3- and 4-CQA [19,20,36]. Besides isomerization, there is a possibility that the cysteine may bind to two different sites in 3-, 4-, or 5-CQA, explaining the six Cys-CQA adducts. This phenomenon was furthermore investigated by following the formation of the adducts at different pH values and assessing whether the amino or thiol group of cysteine is involved in the covalent bond. Li et al. [37] showed that the reaction between thiol groups and quinones is kinetically more favored than the reaction between amino groups and quinones. However, Poojary et al. [24] found only one single peak in a chromatogram, showing that only one CQA-cysteine adduct was formed while, in the present study, up to six reaction products could be separated and identified. Moreover, the only adduct identified by Poojary et al. yielded a *m*/*z* of 474, while in the present investigations, the precursor was *m*/*z* 476. The reason for these differences could lie in the different types of sample preparation (esp. with regard to the oxidation, pH, and mass spectrometric analysis conditions applied). A recent study by Drucker et al. [38] also proposed two CQA-cysteine adducts with structures showing *m*/*z* 474 and *m*/*z* 476, thus justifying the observations made in the current study. For the *m*/*z* 476 adduct observed under alkaline conditions, different structures are theoretically possible (Figure 5(aI,aII)), whereby the proposed structure II by Drucker et al. (2023) [38] seems to be a more likely candidate, especially since it delivers the same fragmentation pattern during the mass spectrometry analysis as observed in the present study. Nevertheless, this still remains to be reinforced/justified by further complementary NMR analysis.

A peak with *m*/*z* 707 was also observed and allocated to a CQA dimer. Three peaks were detected in the chromatogram, which corresponded to the isomerized and dimerized monomers of *m*/*z* 353 (i.e., of the 3-, 4-, and 5-CQA). Similarly, Fernández-Poyatos et al. [39] showed deprotonated molecular ions at *m*/*z* 707 and 353 and fragmentation characteristics of CQA, thus confirming this statement. The resulting fragmentation pattern, together with the optimized collision energies used for the MS analysis, is provided in Table (Table S5, Supplementary Data).

### 3.3. Effect of pH on Cys-CQA Adduct Formation

Figure 5a gives an overview of the observed reactions and the monitored molecular entities. At pH 2 and pH 12, no adduct could be detected, showing that the adduct-forming reaction at an extreme pH did not take place or may have progressed to a more advanced, most probably polymerized, stage (Figure 5b). At pH 5, the reaction started, and a single signal was obtained from Cys-5CQA. At pH 6, CQA isomerization was initiated (Figure 5c) and increased with an increasing pH, leading to the formation of adducts with 5-CQA, as well as 4- and 3-CQA. The most pronounced Cys-CQA adduct formation was detected at pH 9. When increasing the pH into the basic range, the formation of adducts decreased further again. The formation of the corresponding di-Cys-CQA adducts showed a comparable behavior (Figure S8A). However, it is possible that the dimers were not a product of two isomerized Cys-CQA adducts but rather a phenomenon observed due to ion-clustering.

The corresponding cysteine blanks (Figure 5d) indicate the presence of the oxidized derivative of cystine, which decreased again at higher values (pH 9). Similar patterns of cysteine/cystine were observed in the 1:1 mixture with CQA (Figure S8B). The peak area for cysteine decreased at a higher pH value, and cysteine reached its highest dimerization at pH 7.

Figure 5c documents the results for CQA blanks with the deprotonated molecular ion species at *m*/*z* 707 (dimer) and 353 (monomer), as well as strong isomerization at pH 9, which is in line with the highest amount of Cys-CQA adduct formation (Figure 5b). While monitoring the 5-CQA content in a 1:1 mixture with cysteine (Figure S8C), it was also observed that in acidic solutions, only initial 5-CQA and its dimer were present. No isomerization of 5-CQA had taken place. However, this isomerization already began in the neutral pH range (pH 6 and 7), and 4-CQA, as well as its dimer, was formed. Under basic conditions, 3-CQA and its dimer were also formed (Figure S8C). The signal of all CQA forms increased up to pH 11 and then started to decrease again. Wang et al. [36] also documented that the stability of 5-CQA and its isomers gradually decreased with increasing pH.

**Figure 6:**
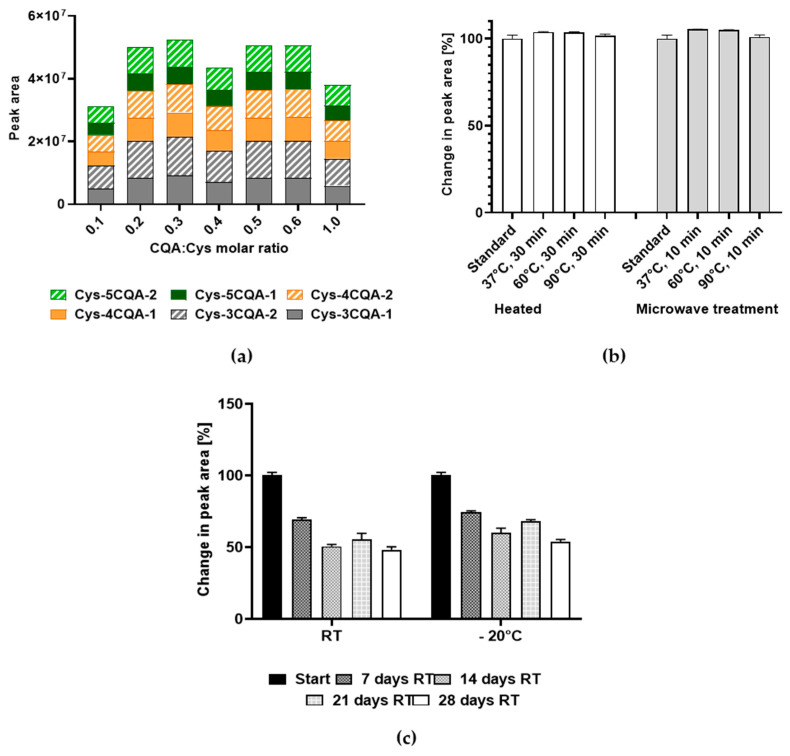
Concentration dependence of the adduct formation and their stability. (**a**) Effect of reactant ratios on adduct formation; (**b**) effect of heat and microwave treatment on the Cys-CQA adducts; and (**c**) effect of long-term storage on Cys-CQA adducts. Abbreviations: CQA—caffeoylquinic acid, Cys—cysteine, and RT—room temperature. Digits 1 and 2 in (**a**) correspond to the two possible position isomers.

Different Cys-CQA molar ratios were further assessed at pH 9 (Figure S9). The Cys-CQA molar ratio of 0.3 tended to be the most suitable for forcing adduct formation, where a lower concentration of one of the reactant molecules corresponded to a lower adduct formation (Figure 6a). Equimolar concentrations of both constituents also led to the lower formation of the adducts. All the other monitored molecules are illustrated in Figure S9. The composition of the six possible Cys-CQA adducts formed remained consistent (Figure 6a). These results confirm the chosen optimum pH 9 using the mixture of 30 mM 5-CQA and 100 mM cysteine (ratio 1:1, *v*/*v*), which were incubated for 20 h. When one considers the concentration ratios of CQA to the cysteine used a positive correlation to the adduct formation of both molecules can possibly be derived, depending on the concentration used. The maximum CQA concentration used in these experiments was 30 mM, as it was already at the limit of its solubility in water. It could then be possible to achieve higher adduct formation using a different solvent in which CQA is more soluble.

Figure 6b shows the change in the peak areas of the CQA-cysteine adducts of *m*/*z* 476 depending on the heat or microwave treatment at a defined temperature (37, 60, and 90 °C) compared to normal incubation at room temperature only. The results of the other molecular entities monitored can also be found in Figure S10. Generally, the values fluctuate for all the molecules approximately in the range of 98–110%, with a slight increase after the treatments at 37 and 60 °C, returning to slightly lower values after treatment at 90 °C. The reason for these observed slight changes could lie in the better solvation of the samples at comparatively higher temperatures and/or eventually better ionization during HPLC-MS/MS analysis. An exception has to be made here for cysteine, which oxidizes to cystine under the conditions applied. Subsequently, higher amounts were recorded, especially at 60 and 90 °C (Figure S10C,D).

Figure 6c shows the change in the CQA-cysteine adducts of the *m*/*z* 476 as a function of storage at room temperature or at −20 °C over a period of 28 days compared to direct measurement by HPLC-MS/MS. The results for the other molecules monitored are depicted in Figure S11. In general, the amounts of total CQA (3-,4-, and 5-CQA, *m*/*z* 353), their dimers (*m*/*z* 707), as well as cysteine and their adducts (*m*/*z* 476/951) decreased over the storage time at room temperature and at −20 °C. An exception is noted for the total CQA, where after 28 days, the values rose again. Again, cysteine oxidized to produce pronounced amounts of cystine (Figure S11D). The monitored substances tended to be more stable when stored at −20 °C than at room temperature. The observed decrease was likely caused by further reactions involving eventually both degradation and/or further polymerization processes. This aspect will be subject to further investigations in the future.

Finally, the effect of a strong thiol-free reducing agent tris(2-carboxyethyl)phosphine (TCEP) that maintains high activity at a low pH was investigated to check the stability of the Cys-CQA adducts. The results are summarized in Figure S12. The total amounts of cysteine, cystine and the two adducts at *m*/*z* 476 and 951 showed a strong reduction after this treatment indicating eventually a further complexation, especially since the amount of cysteine also decreased and was not released either from cystine or their adducts, whereas the amount of total CQA (3-,4-, and 5-CQA, *m*/*z* 353) and their dimers (*m*/*z* 707) remained more or less constant (Figure S12). These data suggest that the adducts are liable to reduction or complexation with TCEP. Further experiments with alternative reducing agents, while simultaneously applying thiol blocking agents, are necessary to address these observations more specifically and to shed more light on the reducibility of the Cys-CQA adducts. In the same context, Jongberg et al. (2015) investigated the adduct formation of β-lactoglobulin with quinones and their subsequent reduction by TCEP and other reducing agents. In their study, the protein adducts also dissociated when TCEP was used [40]. These and our results document that during sample preparation for the analysis of proteins, disulfide bonds may not only be reduced but also a significant proportion of the protein-polyphenol adducts may be decomposed.

### 3.4. Formation of CQA Adducts with Other Thiol-Containing Reactants

**Figure 7:**
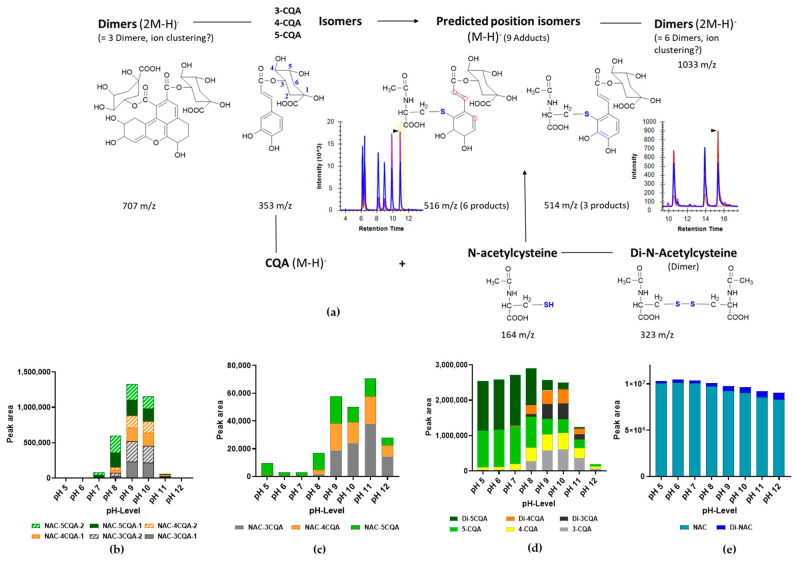
Reaction of CQA with NAC, as affected by pH. (**a**) Major reaction products were monitored, and the red rings indicate possible reaction sites; (**b**) effect of pH on adduct *m*/*z* 516 formation; (**c**) effect of pH on adduct *m*/*z* 514 formation; (**d**) iso- and dimerization observed for CQA; and (**e**) observed dimerization of cysteine. Digits 1 and 2 in (**b**) correspond to the two possible position isomers.

Experiments with NAC and CQA were conducted to confirm that the six observed adducts described above resulted only from the participation of the thiol group of cysteine, as the free amino group of cysteine was blocked. Figure 7a gives an overview of the observed reactions and the monitored molecular entities at different pH conditions while using the mixture of 30 mM 5-CQA and 100 mM NAC with a ratio of 1:1 (*v*/*v*) when incubated for 20 h. This method investigated the individual substances of the reaction, including three isomers of CQA (*m*/*z* 353) with its dimer (*m*/*z* 707) as well as NAC (*m*/*z* 164) with its corresponding dimer (*m*/*z* 323) and the adducts likely to be formed (*m*/*z* 516 or 514) with the adduct dimer (*m*/*z* 1033). The molecule with *m*/*z* 514 is an adduct that contains three double bonds in the benzene ring in contrast to the adduct with *m*/*z* 516 (Figure 7a). As expected, six peaks corresponding to the formation of NAC-CQA adducts with *m*/*z* 516 were obtained, which, as with the reaction of cysteine and CQA, can be attributed to the three 5-, 4-, and 3- CQA isomers. Thus, it can be further assumed that the adducts of NAC formed with chlorogenic acid are probably also positional isomers after the isomerization of 5-CQA to 3- and 4-CQA (*m*/*z* 353). Here, the amount of adduct initially increased to reach its maximum at pH 9 before starting to decrease again (Figure 7b—*m*/*z* 516/Figure S13A—*m*/*z* 1033). In contrast to the cysteine reaction, however, the reaction with NAC showed that two adduct types of different molecular sizes were formed (*m*/*z* 516 and 514). When the course of the adduct distribution at *m*/*z* 516 was still similar to that of the cysteine adduct distribution, as described above, the NAC-CQA adducts with the lower molecular mass of *m*/*z* 514 showed a different distribution (Figure 7c). Here, the amount of adduct initially increased until pH 9. The chromatogram also showed a different number of peaks for the adduct occurrence. While there were still six peaks with *m*/*z* 516, as with the cysteine-CQA adduct, only three peaks were detected when looking at *m*/*z* 514 (Figure 7c). It still remains unclear why this occurs during CQA adduct formation with NAC but not in combination with pure cysteine. Further experiments should be carried out to clarify this observation. Furthermore, this phenomenon also explains the deviation from the descriptions by Poojary et al. [24] on the adduct formation of cysteine with CQA, where three double bonds were apparently also present in the benzene ring. Eventually, stronger oxidation when using periodate for the oxidation of CQA and adduct formation may explain these observations, but this aspect needs to be confirmed. Altogether, an optimum pH of 9 for incubation of the standard solutions can also be confirmed for adduct formation with NAC, as both adducts with *m*/*z* 516 and 514 reached their maximum at this pH.

Figure 7d documents the results of CQA in the reaction mixture with NAC with the deprotonated molecular ion species at *m*/*z* 707 (dimer) and 353 (monomer), as well as strong isomerization at pH 9 in line with the results observed during cysteine modification. A similar trend was also observed in the CQA blank solutions (Figure S13B). The corresponding NAC monitoring in the 1:1 mixture with CQA indicates the presence of the oxidized derivative of the amino acid with increasing pH (Figure 7e). A similar pattern of NAC oxidation was also observed in the corresponding NAC blank solutions (Figure S13C).

**Figure 8:**
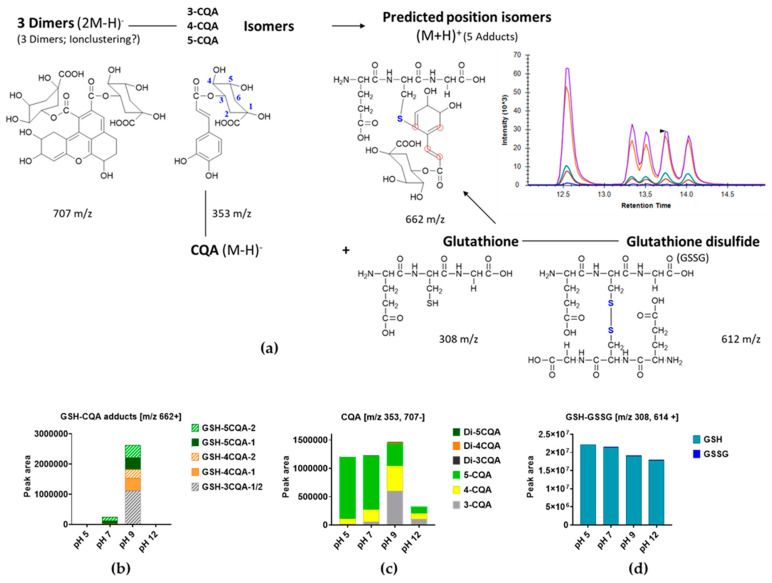
Reaction of CQA with GSH, as affected by pH. (**a**) Major reaction products were monitored, and the red rings indicate possible reaction sites; (**b**) effect of pH on adduct *m*/*z* 662 formation; (**c**) iso- and dimerization observed for CQA; and (**d**) observed dimerization of GSH to GSSG. Abbreviations: CQA—caffeoylquinic acid, GSH—glutathione, and GSSG—oxidized glutathione. Digits 1 and 2 in (**b**) correspond to the two possible position isomers.

Going a step further, glutathione, a small peptide with a molar mass of 307.33 g/mol that occurs ubiquitously in plant cells, was allowed to interact with CQA. The aim of this set of experiments was to observe how the adduct formation of chlorogenic acid with peptides behaves and whether the comparatively larger molecular size changes with the reaction process due to steric hindrance. For this purpose, mixtures of 5-CQA and glutathione were also incubated at different pH values in order to allow the formation of adducts. It was assumed that the reaction would proceed similarly to the reaction with cysteine. In total, five different peaks were identified as adduct products. It was assumed that a further peak could be worked out with better separation of the position of the isomers of the 3-CQA adduct, as the height of the first peak suggested that it contained more than one component (Figure 8a). The present study also characterized the individual substances of the reaction, with three isomers of CQA (353 *m*/*z*; dimer 707 *m*/*z*) and glutathione (308 *m*/*z*; dimer 612 *m*/*z*), as well as the adducts expected to form with *m*/*z* 662. The evaluation here also shows a similar distribution of individual substances and the analysis of the cysteine-CQA adducts. Adduct formation was analyzed only at selected pHs 5, 7, 9, and 12 based on the foregoing results. In acidic (pH 5) and strongly basic (pH 12) solutions, no adduct product could be observed. Isomerization began at pH 7, and glutathione adducts with 4- and 5-CQA were found, with incubation at pH 9, producing the highest amounts of the GSH-3-CQA adduct (Figure 8b).

Figure 8c documents the results for CQA in the reaction mixture for the deprotonated molecular ion species at *m*/*z* 707 (dimer) and 353 (monomer), as well as strong isomerization at pH 9 in line with the results observed during cysteine modification (Figure 8c). The corresponding GSH monitoring in the mixture with CQA (Figure 8d) confirmed the presence of the oxidized derivative of the peptide with increasing pH. A similar pattern of GSH oxidation was also observed in the corresponding GSH blank solutions. The position isomers (*m*/*z* 662) observed during the reaction of glutathione with CQA under alkaline conditions have also been reported [38], with only three dominant products identified, including at least the presence of three other *m*/*z* 662 adducts with much lower abundances. The same authors also reported a partly similar fragmentation pattern during the mass spectrometry analysis, as observed in the present work. The question still remains as to how large the reacting peptide needs to be before it causes steric hindrance. Further experiments are being conducted to follow-up on this issue while characterizing the CQA modifications in the coffee storage protein, as recently published [41].

### 3.5. Identification of Cysteine-Chlorogenic Acid Adducts in Coffee Samples

**Figure 9:**
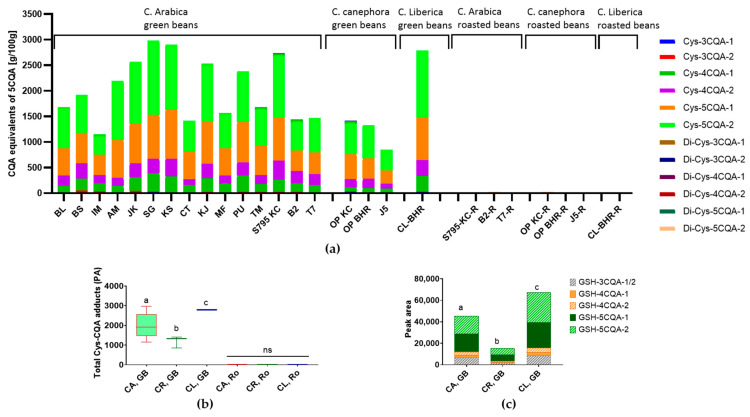
Identification of thiol-CQA adducts in coffee beans. (**a**) Major Cys-CQA reaction products found in green coffee beans; (**b**) comparison of the total peak area of Cys-CQA adducts in coffee beans; and (**c**) comparison of GSH-CQA adducts in coffee beans. CQA—caffeoylquinic acid, Cys-cysteine, GSH—glutathione, CA = *C. arabica*; CR = *C. canephora*; CL = *C. liberica*; GB = green beans; and Ro = roasted. Different letters mark significant differences (*p* < 0.05), ns: no significant. Digits 1 and 2 in (**a**,**c**) correspond to the two possible position isomers.

Following the experiments on adduct formation between chlorogenic acid and cysteine, the content of Cys-CQA adducts in the coffee samples was investigated. Rao & Fuller (2018) described an approximate pH value of five as typical for coffee [42]. Therefore, similarities to the preliminary investigation of the reaction of CQA with cysteine at pH 5 were expected. Due to the lack of data, the results can only be compared with model experiments, as corresponding studies dealing with the formation of Cys-CQA adducts in coffee beans are not available. Therefore, the linearity was performed with different amounts of the adducts (Figure S14, Supplementary Data), and the limits of detection (LOD) and quantification (LOQ) were calculated. Data obtained for the green and roasted coffee beans are illustrated in Figure 9a. Preliminary studies on cysteine-CQA adduct formation at pH 5 showed only cysteine adducts with 5-CQA. However, the adduct analysis in the coffee matrix revealed the presence of Cys-4CQA adducts in addition to the Cys-5CQA adducts being mainly present. Cys-3CQA adducts and Cys-CQA dimers were also found, but these were only rarely present. In the preliminary study, however, isomerization only began at pH 7, which is why Cys-4CQA adducts were only detectable at this pH value. This shows the influence of the coffee matrix on the isomerization and dimerization of the coffee’s own chlorogenic acids. What exactly in the coffee bean is responsible for the isomerization of the chlorogenic acid must be narrowed down by further investigations. In the experiments conducted here, green coffee samples of the *Arabica* varieties showed significantly higher adduct contents (*p* < 0.001) than those of the *Canephora* and *Liberica* varieties (Figure 9b).

In the same context, we could also identify for the first time the previously described GSH-CQA adducts in the coffee beans similar to the pattern obtained for the preliminary model studies on GSH-CQA adduct formation at pH 9 (Figure 8). The composition increased in the order GSH-5CQA >> GSH-3CQA > GSH-4CQA. Again, *Arabica* and *Liberica* varieties showed significantly (*p* < 0.05) higher adduct contents than those of the *Canephora* variety, although the latter did contain higher amounts of chlorogenic acids.

Poojary et al. [24] also investigated the adduct formation of cysteine and CQA in milk-containing coffee beverages, and they were already able to confirm the covalent bonds between the milk proteins and chlorogenic acids. They were also able to detect traces of cysteine-CQA adducts in the beverages. These findings confirm the results of this work in that adducts of cysteine and chlorogenic acids may be present in coffee-containing samples.

While the dimer compounds of cysteine-5CQA adducts were found in traces (Di-Cys-5CQA), hardly any Cys-CQA adducts and no GSH-CQA adducts could be detected in the corresponding roasted coffee bean samples. Thus, the adducts may no longer be present due to conversion during roasting. CQAs are aroma precursors, and high temperatures cause the organic components of the coffee beans to decompose, and/or they are consumed/integrated into the course of the Maillard reaction to form melanoidins. Further work is necessary to follow-up on the fate of such thiol-containing adducts during roasting.

## 4. Conclusions

In this work, the presence of adducts formed by phenolic compounds found in abundance in coffee beans was explored. Particular emphasis was placed on the analysis of adducts between chlorogenic acid and cysteine. The primary characterization of the different coffee samples showed a different composition in terms of dry matter, raw protein content, antioxidant capacity, caffeine, free amino acids, and chlorogenic acid content in the different coffee varieties (*C. arabica*, *C. canephora*, and *C. liberica*). Chlorogenic acid distribution showed that 5-CQA was the major component in both green and roasted coffee beans. Methods based on targeted mass spectrometry were efficiently developed to analyze the adducts formed, initially using model solutions of cysteine and 5-CQA. The results showed a total of six reaction products between CQA and cysteine, with the optimum reported at pH 9, demonstrating both the possibility of the isomerization of 5-CQA to 3- and 4-CQA in an alkaline medium and the possibility of cysteine binding to two potential sites of the three isomers. Further experiments revealed the presence of six adducts with N-acetyl-cysteine, while glutathione produced five adducts, and a sixth adduct with the positional isomers of 3-CQA could be elaborated with a more efficient separation. This extended analysis highlighted that cysteine binds only with its thiol group to two possible sites—in the structure of caffeoylquinic acid—and that its basic amino group (-NH2) was not involved in the reaction. Finally, Cys-CQA adducts were identified and relatively quantified in the coffee samples, with Cys-4CQA and Cys-5CQA as the main adducts in the extracts. In the same context, it was also possible to identify in coffee samples for the first time adducts of chlorogenic acid with glutathione (GSH-5CQA, GSH-3CQA, and GSH-4CQA). Given that other amino acids and chlorogenic acid derivatives present in green coffee beans could also participate in similar reactions for the formation of further complex crosslinks, more detailed investigations in this field were the subject of follow-up projects. In addition, proteins and, in particular, the main storage protein 11S can also be involved in similar reactions with chlorogenic acid, and this represents a further area of research while applying a proteomics-based strategy. Furthermore, these results indicate that post-harvest processing could play a vital role in the progress of such reactions and needs to be followed up in future studies. Finally, two aspects need future attention: The first one should encompass the resorption of such adducts in organisms and their in vitro/in vivo biological activity, where the presence of an amino component in the adducts could eventually better facilitate intake via active transport. The second venue of research lies in the fate of such adducts during further coffee processing, i.e., roasting and the eventual contribution to taste and formation of more complex browning products.

## Figures and Tables

**Table 1:** Characteristics of the green and roasted coffee bean samples.

	Coffee Samples	Dry Matter (g/100 g)	Antioxidant Capacity (g/100 g)	Protein Content (g/100 g)	Caffeine Content	
					UV (g/100 g)	MS (g/100 g)
**Green coffee beans**	BL	91.14 ± 0.16 ^a^	3.03 ± 0.19 ^a^	13.29 ± 0.06 ^a^	0.826 ± 0.001 ^a^	1.337 ± 0.003 ^a^
	BS	91.36 ± 0.02 ^a,b^	3.02 ± 0.03 ^a^	13.62 ± 0.09 ^a^	0.593 ± 0.002 ^a^	0.967 ± 0.001 ^a^
	IM	91.81 ± 0.01 ^b^	2.95 ± 0.26 ^a^	12.68 ± 0.16 ^b^	0.684 ± 0.004 ^a^	1.206 ± 0.003 ^a^
	AM	91.35 ± 0.14 ^a,b^	3.12 ± 0.10 ^a^	11.28 ± 0.05 ^c^	0.520 ± 0.002 ^a^	0.856 ± 0.003 ^b^
	JK	91.52 ± 0.02 ^a,b^	2.91 ± 0.20 ^a^	12.31 ± 0.10 ^d^	0.702 ± 0.008 ^a^	1.142 ± 0.001 ^a,b^
	SG	91.07 ± 0.03 ^a^	3.28 ± 0.21 ^b^	12.34 ± 0.08 ^d^	0.619 ± 0.000 ^a^	1.014 ± 0.001 ^a,b^
	KS	90.94 ± 0.04 ^a^	3.00 ± 0.15 ^a^	12.39 ± 0.02 ^b,d^	0.576 ± 0.000 ^a^	0.998 ± 0.008 ^a,b^
	CT	90.01 ± 0.09 ^c^	2.36 ± 0.18 ^c^	12.35 ± 0.08 ^b^	0.635 ± 0.003 ^a^	1.040 ± 0.005 ^a,b^
	KJ	90.04 ± 0.02 ^c^	3.29 ± 0.19 ^b^	11.49 ± 0.02 ^c^	0.688 ± 0.004 ^a^	1.093 ± 0.002 ^a,b^
	MF	91.03 ± 0.02 ^a,d^	2.54 ± 0.06 ^c^	11.94 ± 0.20 ^e^	0.538 ± 0.00 ^a^	0.961 ± 0.008 ^a,b^
	PU	91.06 ± 0.06 ^a^	2.56 ± 0.07 ^c^	12.57 ± 0.18 ^b^	0.651 ± 0.001 ^a^	1.058 ± 0.004 ^a,b^
	TM	90.30 ± 0.01 ^c,e^	2.71 ± 0.13 ^c^	11.72 ± 0.08 ^e^	0.531 ± 0.002 ^a^	0.865 ± 0.006 ^b^
	S795 KC	92.84 ± 0.23 ^f^	2.71 ± 0.04 ^c^	12.72 ± 0.17 ^b^	0.543 ± 0.002 ^a^	0.750 ± 0.002 ^b^
	B2	90.59 ± 0.04 ^d,e^	2.18 ± 0.05 ^c^	13.11 ± 0.11 ^a^	0.785 ± 0.000 ^a^	0.794 ± 0.007 ^b^
	T7	92.02 ± 0.01 ^b^	2.50 ± 0.04 ^c^	13.09 ± 0.16 ^a^	0.859 ± 0.002 ^a^	0.887 ± 0.003 ^b^
	OP KC	92.56 ± 0.34 ^f^	3.39 ± 0.13 ^b^	15.87 ± 0.22 ^f^	0.857 ± 0.005 ^a^	1.515 ± 0.051 ^a^
	OP BHR	93.16 ± 0.15 ^f^	3.23 ± 0.03 ^a,b^	16.20 ± 0.25 ^f^	1.153 ± 0.007 ^a^	1.792 ± 0.014 ^c^
	J5	88.89 ± 0.08 ^g^	2.77 ± 0.26 ^a,d^	15.32 ± 0.01 ^g^	0.944 ± 0.001 ^a^	0.965 ± 0.006 ^a,b^
	CL-BHR	93.17 ± 0.14 ^f^	2.32 ± 0.16 ^c^	12.72 ± 0.43 ^b^	0.537 ± 0.002 ^a^	0.804 ± 0.006 ^b^
**Roasted coffee beans**	S795-KC-R	95.83 ± 0.14 ^h^	2.75 ± 0.09 ^a,d^	12.88 ± 0.24 ^a,b^	0.633 ± 0.001 ^a^	0.965 ± 0.004 ^a,b^
	B2-R	98.65 ± 0.05 ^i^	3.13 ± 0.30 ^a,b^	13.18 ± 0.04 ^a^	1.389 ± 0.003 ^b^	1.428 ± 0.001 ^a,c^
	T7-R	95.64 ± 0.06 ^h^	2.53 ± 0.07 ^c,d^	13.55 ± 0.10 ^a^	1.309 ± 0.009 ^b^	1.344 ± 0.001 ^a,b^
	OP KC-R	96.26 ± 0.20 ^j^	3.58 ± 0.12 ^b^	16.40 ± 0.59 ^f^	1.053 ± 0.002 ^a,b^	1.659 ± 0.010 ^a,c^
	OP BHR-R	95.94 ± 0.12 ^h^	3.34 ± 0.21 ^a,b^	16.29 ± 0.27 ^f^	1.111 ± 0.002 ^a,b^	1.557 ± 0.035 ^a,c^
	J5-R	98.07 ± 0.11 ^k^	4.31 ± 0.18 ^e^	15.60 ± 0.02 ^g^	2.549 ± 0.009 ^c^	2.572 ± 0.010 ^d^
	CL-BHR-R	95.96 ± 0.24 ^h,j^	3.41 ± 0.25 ^a,b^	12.77 ± 0.29 ^b^	0.643 ± 0.003 ^a^	0.859 ± 0.002 ^b^

The data are expressed as the mean ± standard deviation, *n* = 3. Different letters within columns indicate significantly different values (*p* < 0.05).

## Data Availability

The original contributions presented in the study are included in the article and Supplementary Materials, further inquiries can be directed to the corresponding author.

## Supplementary Materials

The following supporting information can be downloaded at: https://www.mdpi.com/article/10.3390/foods13111660/s1, Table S1. Identification of chlorogenic acid and related hydroxycinnamic acids; Table S2. Coffee bean samples of different origins and processing; Table S3. HPLC–MS/MS conditions for separation and determination of the free amino acids in the coffee bean extracts; Table S4. HPLC–MS/MS conditions for separation and characterization of the chlorogenic acids (CQAs) and caffeine; Table S5. HPLC–MS/MS conditions for separation and characterization of the cysteine-chlorogenic acid adducts (Cys-CQA); Table S6. HPLC–MS/MS conditions for separation and characterization of the N-acetyl-cysteine-chlorogenic acid adducts (NAC-CQA); Table S7. HPLC–MS/MS conditions for separation and characterization of the glutathione-chlorogenic acid adducts (GSH-CQA); Figure S1. Dry matter of the coffee samples; Figure S2. Protein content according to Kjeldahl in the coffee varieties; Figure S3. Antioxidant capacity of the coffee samples; Figure S4. Caffeine content of the individual samples as measured by UV (280 nm) and MS/MS (MRM); Figure S5. Chromatograms of the analyzed compounds, above (UV 325 nm), followed by UV measurement at 280 nm, and finally the TIC (total ion chromatograms) from MS/MS data; Figure S6. Chlorogenic acid content of the individual samples, as measured by UV (325 nm) and MS/MS (MRM); Figure S7. Identification of Cys-CQA adducts. A: Superimposed chromatograms of the MS scans of the three standard solutions with 30 mM chlorogenic acid, 100 mM cysteine, or their 1:1 mixture; colors: green cysteine + chlorogenic acid; Figure S8. Chromatograms and amounts (peak area) of reaction products occurring during the incubation of CQA and Cys at different pH conditions with 30 mM chlorogenic acid and 100 mM cysteine in their 1:1 mixture; Figure S9. Concentration dependence of the adduct formation after incubation for 20 h at different concentration ratios of the reactant molecules mixed 1:1 (*v*/*v*) at the optimum pH of 9 and at room temperature; Figure S10. Stability of the adducts after incubation of 30 mM chlorogenic acid and 100 mM cysteine in their 1:1 mixture for 20 h at the optimum pH 9 and at room temperature. The mixtures were acidified to stop their reaction; Figure S11. Long-term effect of storage of the adducts at room temperature (RT) and −20 °C after incubation of 30 mM chlorogenic acid and 100 mM cysteine in their 1:1 mixture for 20 h at the optimum pH 9 and at room temperature; Figure S12. Effect of incubation with TCEP on the monitored compounds as percentage change compared to samples to which no TCEP was added (Standard); Figure S13. Amounts (peak area) of reaction products occurring during the incubation of CQA and NAC at different pH conditions with 30 mM chlorogenic acid and 100 mM NAC in their 1:1 mixture; Figure S14. Linearity for the MS response for different amounts of the reaction products produced during the incubation of CQA and Cys at pH 9 with 30 mM chlorogenic acid and 100 mM Cys in their 1:1 mixture. Abbreviations: CQA—caffeoylquinic acid, Cys—cysteine, Di-CQA—dimer of caffeoylquinic acid, and MS—mass spectrometry.
