# Supplementary material for: Formation of Cysteine Adducts with Chlorogenic Acid in Coffee Beans
Source: Foods. 2024 May 25;13(11):1660. doi: 10.3390/foods13111660 (PMC11171587; doi:10.3390/foods13111660)
Supplement: Supplementary file 1 [file foods-13-01660-s001.zip › foods-3007733-supplementary.pdf]

Supplementary Material

Formation of cysteine adducts with chlorogenic acid in coffee beans

Sorel Tchewonpi Sagu [1], Nina Ulbrich [2,3], Johanna Rebekka Morche [2], Kapil Nichani [2], Haydar Özpinar [4], Steffen Schwarz [5], Andrea Henze [1], Sascha Rohn [3] and Harshadrai M. Rawel [2,*]

[1] Institute of Agricultural and Nutritional Sciences, Martin Luther University Halle-Wittenberg, Von-Danckelmann-Platz 2, 06120 Halle (Saale), Germany; sorel.sagu@landw.uni-halle.de (S.T.S.); andrea.henze@landw.uni-halle.de (A.H.)

[2] Institute of Nutritional Science, University of Potsdam, Arthur-Scheunert-Allee 114-116, 14558 Nuthetal, Germany; nina.ulbrich@tu-berlin.de (N.U.); johanna.rebekka.morche@uni-potsdam.de (J.R.M.); kapil.nichani@uni-potsdam.de (K.N.)

[3] Institute of Food Technology and Food Chemistry, Technische Universität Berlin, Gustav-Meyer-Allee 25, 13355 Berlin, Germany; rohn@tu-berlin.de

[4] Department of Nutrition and Dietetics, Faculty of Health Sciences, İstanbul Aydın Üniversitesi, Mah. İnönü Cad. No: 38 Sefaköy, 34295 İstanbul, Turkey; haydarozpinar@aydin.edu.tr

[5] Coffee Consulate, Hans-Thoma-Strasse 20, 68163 Mannheim, Germany; schwarz@coffee-consulate.com

* Correspondence: rawel@uni-potsdam.de; Tel.: +49-332-0088-5525

Content

Table S1. Identification of Chlorogenic acid and related hydroxycinnamic acids

Table S2. Coffee bean samples of different origin and processing

Table S3. HPLC – MS/MS conditions for separation and determination of the free amino acids in the coffee bean extracts

Table S4. HPLC – MS/MS conditions for separation and characterization of the chlorogenic acids (CQAs) and caffeine

Table S5. HPLC – MS/MS conditions for separation and characterization of the cysteine-chlorogenic acid-adducts (Cys-CQA)

Table S6. HPLC – MS/MS conditions for separation and characterization of the N-acetyl-cysteine-chlorogenic acid-adducts (NAC-CQA)

Table S7. HPLC – MS/MS conditions for separation and characterization of the glutathione-chlorogenic acid-adducts (GSH-CQA)

Figure S1. Dry matter of the coffee samples

Figure S2. Protein content according to Kjeldahl in the coffee varieties

Figure S3. Antioxidant capacity of the coffee samples

Figure S4. Caffeine content of the individual samples as measured by UV (280 nm) and MS/MS (MRM).

Figure S5. Chromatograms of the analyzed compounds, above (UV 325 nm), followed by UV measurement at 280 nm and finally the TIC (Total ion Chromatograms) from MS/MS Data.

Figure S6. Chlorogenic acid content of the individual samples as measured by UV (325nm) and MS/MS (MRM).

Figure S7. Identification of Cys-CQA adducts. A: Superimposed chromatograms of the MS scans of the three standard solutions with 30 mM chlorogenic acid, 100 mM cysteine or their 1:1 mixture; colors: green - cysteine + chlorogenic acid

Figure S8. Chromatograms and amounts (peak area) of reaction products occurring during the incubation of CQA and Cys at different pH conditions with 30 mM chlorogenic acid and 100 mM cysteine in their 1:1 mixture

Figure S9. Concentration dependence of the adduct formation after incubation for 20h at different concentration ratios of the reactant molecules mixed 1:1 (v/v) at the optimum pH 9 and at room temperature

Figure S10. Stability of the adducts after incubation of 30 mM chlorogenic acid and 100 mM cysteine in their 1:1 mixture for 20h at the optimum pH 9 and at room temperature. The mixtures were acidified to stop their reaction

Figure S11. Long-term effect of storage of the adducts at room temperature (RT) and -20°C after incubation of 30 mM chlorogenic acid and 100 mM cysteine in their 1:1 mixture for 20h at the optimum pH 9 and at room temperature.

Figure S12. Effect of incubation with TCEP on the monitored compounds as a percentage change compared to samples to which no TCEP was added (Standard)

Figure S13. Amounts (peak area) of reaction products occurring during the incubation of CQA and NAC at different pH conditions with 30 mM chlorogenic acid and 100 mM NAC in their 1:1 mixture.

Figure S14. Linearity for the MS response for different amounts of the reaction products produced during the incubation of CQA and Cys at pH 9 with 30 mM chlorogenic acid and 100 mM Cys in their 1:1 mixture; abbreviations: CQA - caffeoylquinic acid, Cys - cysteine, Di-CQA - dimer of caffeoylquinic acid, MS - Mass spectrometry.

Table S1. Identification of Chlorogenic acid and related hydroxycinnamic acids*

Name and Abbreviation	Abbreviation	R3	R4	R5	PubChem CID	Comments
3-*O*-caffeoylquinic	3-CQA	C	H	H	1794427	Syn: Chlorogenic acid
4-*O*-caffeoylquinic	4-CQA	H	C	H	9798666	Syn: Cryptochlorogenic acid
5-*O*-caffeoylquinic	5-CQA	H	H	C	5280633	Syn: Neochlorogenic acid: Main component in Coffee/Apple.
3-*O*-*p*-coumaroylquinic acid	3-*p*CoQA	*p*-Co	H	H	9945785	
4-*O*-*p*-coumaroylquinic acid	4-*p*CoQA	H	*p*-Co	H	5281766	
5-*O*-*p*-coumaroylquinic acid	5-*p*CoQA	H	H	*p*-Co	6441280	
3-*O*-feruloylquinic acid	3-FQA	F	H	H	10133609	
4-*O*-feruloylquinic acid	4-FQA	H	H	F	10177048	
5-*O*-feruloylquinic acid	5-FQA	H	F	H	131853509	
3,4-di-*O*-caffeoylquinic acid	3,4-diCQA	C	C	H	5281780	
3,5-di-*O*-caffeoylquinic acid	3,5-diCQA	C	H	C	6474310	
4,5-di-*O*-caffeoylquinic acid	4,5-diCQA	H	C	C	6474309	
3-*O*-feruloyl, 4-O-caffeoylquinic acid	3F-4CQA	F	C	H	?	
4-*O*-feruloyl, 3-O-caffeoylquinic acid	4F-3CQA	C	F	H	?	
3-*O*-feruloyl, 5-O-caffeoylquinic acid	3F-5CQA	F	H	C	?	
5-*O*-feruloyl, 3-O-caffeoylquinic acid	5F-3CQA	C	H	F	?	
4-*O*-feruloyl, 5-O-caffeoylquinic acid	4F-5CQA	H	F	C	?	
5-*O*-feruloyl, 4-O-caffeoylquinic acid	5F-4CQA	H	C	F	?	

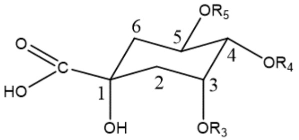

Q

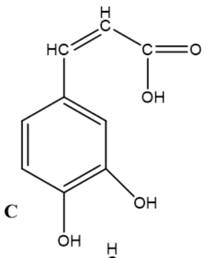

C

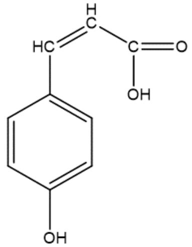

p-Co

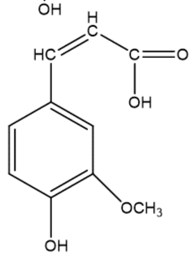

F

* According to Clifford, M.N.; Johnston, K.L.; Knight, S.; Kuhnert, N. Hierarchical scheme for lc-msn identification of chlorogenic acids. J Agric Food Chem 2003, 51, 2900-2911.

Abbreviations	PubChem CID
Syn = Synonyms	
C = Caffeic acid	689043
F = Ferulic acid	445858
p-Co = para Coumaric acid	637542
Q = Quinic acid	6508

Table S2. Coffee bean samples of different origin and processing

Processing	Sample code	Country of origin	Species	Source
Green coffee beans	BL	Brazil (Logoa)	*C. Arabica*	Coffeewell GmbH (Mettmann, Germany)
	BS	Brazil (Santos)	*C. Arabica*	Coffeewell GmbH (Mettmann, Germany)
	IM	India (Malabar)	*C. Arabica*	Coffeewell GmbH (Mettmann, Germany)
	AM	Ethiopia (Maji)	*C. Arabica*	Coffeewell GmbH (Mettmann, Germany)
	JK	Indonesia (Java, Kayumas)	*C. Arabica*	Coffeewell GmbH (Mettmann, Germany)
	SG	Indonesia (Sumatra, Gayo)	*C. Arabica*	Coffeewell GmbH (Mettmann, Germany)
	KS	Colombia (Supremo)	*C. Arabica*	Coffeewell GmbH (Mettmann, Germany)
	CT	Costa Rica (Tarrazu)	*C. Arabica*	Coffeewell GmbH (Mettmann, Germany)
	KJ	Kenya (Josra)	*C. Arabica*	Coffeewell GmbH (Mettmann, Germany)
	MF	Mexico (Flamingo)	*C. Arabica*	Coffeewell GmbH (Mettmann, Germany)
	PU	Peru (Uruba)	*C. Arabica*	Coffeewell GmbH (Mettmann, Germany)
	TM	Tanzania (Mbeya Peak)	*C. Arabica*	Coffeewell GmbH (Mettmann, Germany)
	OP KC1	India (Kerkiecoondah - KC*) OP KC1	*C. Robusta*	Coffee Consulate (68163 Mannheim,
	OP BHR1	India (Balehonnur - BHR*) OP BHR1	*C. Robusta*	Coffee Consulate (68163 Mannheim,
	S795 KC1	India (Kerkiecoondah - KC*) S795 KC31	*C. Arabica*	Coffee Consulate (68163 Mannheim,
	CL BHR1	India (Balehonnur - BHR*) BHR37	*C. Liberica*	Coffee Consulate (68163 Mannheim,
	B2	Turkey- Brazil (Rio Minas)	*C. Arabica*	Prof. Özpinar (Turkey) 2022
	J5	Turkey-India (Cherry AA / AB)	*C. Robusta*	Prof. Özpinar (Turkey) 2022
	T7	Turkey (Highland)	*C. Arabica*	Mehmet Effendi, Prof. Özpinar (Turkey)
Roasted coffee beans	OP KC1-R1	India (Kerkiecoondah - KC*) OP KC1	*C. Robusta*	Coffee Consulate (68163 Mannheim,
	OP-BHR1-R1	India (Balehonnur - BHR*) OP BHR1	*C. Robusta*	Coffee Consulate (68163 Mannheim,
	S795-KC1-R1	India (Kerkiecoondah - KC*) S795 KC31	*C. Arabica*	Coffee Consulate (68163 Mannheim,
	CL-BHR1-R1	India (Balehonnur - BHR*) BHR37	*C. Liberica*	Coffee Consulate (68163 Mannheim,
	B2-R	Brazil (Rio Minas)	*C. Arabica*	Prof. Özpinar (Turkey) 2022
	J5-R	India (Cherry AA / AB)	*C. Robusta*	Prof. Özpinar (Turkey) 2022
	T7-R	Turkey (Highland)	*C. Arabica*	Mehmet Effendi, Prof. Özpinar (Turkey)

BHR - Balehonnur, KC - Kerkiecoondah, OP - Old Paradenia

Table S3. HPLC – MS/MS conditions for separation and determination of the free amino acids in the coffee bean extracts

Sample and Standards

Please see material and methods section.

Internal standard (0.1 mM BOC-Tyr)

External standard fortified with Asn and Gln in the range of 0.1 - 6 µM

HPLC Conditions:

Column: Intrada Amino Acids Column, 150 x 3 mm; 3 µm (Imtakt USA , Portland, USA → MZ-Analysentechnik GmbH, Mainz, Germany); Pressure limits 400 bar; 0.5 ml/min; Column temperature: 35°C

Eluents: A = Acetonitrile (Merck, Darmstadt, Germany) + 0,1 % Formic acid; B = H2O + 50 mM HCOONH4 (Carl Roth GmbH + Co. KG, Karlsruhe, Germany); Flow rate: 0.5 ml/min;

Gradient: 0 min 82 % A; 0-3 min 82% A; 3-10 min 82-80% A; 10-11 min 80-0% A; 11-18 min 0% A; 18-19 min 0-82% A; 19-23 min 82% A; Post run 2 min (Total time 25 min).

Table A1. Elution program

Time [min]	A [%]	B [%]	Flow [mL/min]	Max. Pressure Limit [bar]
0.00	82.0	18.0	0.500	400.00
3.00	82.0	18.0	0.500	400.00
10.00	80.0	20.0	0.500	400.00
11.00	0.0	100.0	0.700	400.00
18.00	0.0	100.0	0.700	400.00
19.00	82.0	18.0	0.500	400.00
23.00	82.0	18.0	0.500	400.00

Sampler: 20 µl

Sampler Temperature: 10 °C

HPLC Sytem: Agilent Infinity 1260 System (binary pump, multicolumn thermostat, vial sampler VL, UV-Vis Dual Wavelength Detector (set at 280/325 nm)

Mass spectrometer: Agilent G6470A Series Triple Quad LC/MS (Agilent Technologies Sales & Services GmbH & Co.KG, Waldbronn, Germany)

MS/MS Conditions:

Collision Gas: Nitrogen

Pressure: 3.41E-5 Torr

Detection: MRM

Fragmentor Voltage: 80 V

Collision Energy (CE, eV) was individually optimized:

Cell Accelator Voltage: 5 kV

Dwell time: 20 ms

ESI Conditions:

Negative Ion-Mode

Temp. sheath gas: 275°C

Sheath Gas: Nitrogen
Flow-Rate (Gas): 11.0 l/min
Capillary (V): 4000 (+Ve); 3500 (-Ve)
Nebulizer pressure: 35.0 psi

Table S3a. MRM Transition list for the measured amino acids

Compound Group	Compound Name	Precursor Ion	Product Ion	Dwell time (ms)	Fragmentor	Collision Energy	Cell Accelerator Voltage	Polarity
	BOC-Tryp	303.2	186	20	60	15	7	Negative
	BOC-Tryp	303.2	141.9	20	60	15	7	Negative
	BOC-Tryp	303.2	100	20	60	15	7	Negative
	BOC-Tyr	280.1	206	20	60	10	7	Negative
	BOC-Tyr	280.1	162.9	20	60	10	7	Negative
	BOC-Tyr	280.1	119	20	60	10	7	Negative
	BOC-Tryp	303.2	229	20	60	15	7	Negative
Ala	Ala	90	55	20	40	15	5	Positive
Ala	Ala	90	44.2	20	40	15	5	Positive
Ala	Ala	90	29.2	20	40	15	5	Positive
Ala	Ala	90	27.1	20	40	15	5	Positive
Arg	Arg	175	116.1	20	40	20	5	Positive
Arg	Arg	175	70.1	20	40	20	5	Positive
Arg	Arg	175	60.1	20	40	20	5	Positive
Asn	Asn	133	87.1	20	40	20	5	Positive
Asn	Asn	133	44.1	20	40	20	5	Positive
Asn	Asn	133	30.2	20	40	20	5	Positive
Asp	Asp	134	116	20	40	10	5	Positive
Asp	Asp	134	88.1	20	40	10	5	Positive
Asp	Asp	134	74.1	20	40	10	5	Positive
Asp	Asp	134	46.1	20	40	10	5	Positive
Cys	Cystein	122.2	76.1	20	80	10	5	Positive
Cys	Cystein	122.2	59.1	20	80	30	5	Positive
Cyt	Cystin	241	122	20	80	15	5	Positive
Cyt	Cystin	241	120	20	80	15	5	Positive
Cyt	Cystin	241	74	20	80	25	5	Positive
Cyt	Cystin	241	152	20	80	10	5	Positive
Glu	Glu	148	102.1	20	40	12	5	Positive
Glu	Glu	148	84.1	20	40	12	5	Positive
Gln	Glutamin	147.1	130	20	60	10	5	Positive
Gln	Glutamin	147.1	84.1	20	60	20	5	Positive
Gln	Glutamin	147.1	56.1	20	60	30	5	Positive
Gln	Glutamin	147.1	41.2	20	60	30	5	Positive
Gly	Gly	76	30.2	20	40	4	5	Positive
Gly	Gly	76	28.2	20	40	4	5	Positive
Gly	Gly	76	48.1	20	40	4	5	Positive
His	His	156	110	20	40	15	5	Positive
His	His	156	95.1	20	40	15	5	Positive
His	His	156	93.1	20	40	15	5	Positive
His	His	156	83.1	20	40	15	5	Positive
Ile	Isoleucin	132.2	86.1	20	60	10	5	Positive
Ile	Isoleucin	132.2	69.2	20	60	20	5	Positive
Ile	Isoleucin	132.2	44.2	20	60	30	5	Positive
Ile	Isoleucin	132.2	30.2	20	60	30	5	Positive

Table S3a. *Cont.*

Compound Group	Compound Name	Precursor Ion	Product Ion	Dwell time (ms)	Fragmentor	Collision Energy	Cell Accelerator Voltage	Polarity
Leu	Leucin	132.2	86.2	20	60	10	5	Positive
Leu	Leucin	132.2	69.2	20	60	20	5	Positive
Leu	Leucin	132.2	44.2	20	60	30	5	Positive
Leu	Leucin	132.2	30.2	20	60	30	5	Positive
Lys	Lysin	147.1	130	20	60	10	5	Positive
Lys	Lysin	147.1	84.1	20	60	20	5	Positive
Lys	Lysin	147.1	56.1	20	60	30	5	Positive
Met	Met	150	133	20	40	11	5	Positive
Met	Met	150	104	20	40	11	5	Positive
Met	Met	150	102.1	20	40	11	5	Positive
Met	Met	150	56.1	20	40	11	5	Positive
Phe	Phe	166	149	20	40	10	5	Positive
Phe	Phe	166	131	20	40	10	5	Positive
Phe	Phe	166	107.1	20	40	10	5	Positive
Phe	Phe	166	103.1	20	40	10	5	Positive
Pro	Pro	116	70.1	20	40	20	5	Positive
Pro	Pro	116	28.2	20	40	20	5	Positive
Ser	Ser	106	88.1	20	40	14	5	Positive
Ser	Ser	106	70.1	20	40	14	5	Positive
Ser	Ser	106	42.1	20	40	14	5	Positive
Thr	Thr	120	103.1	20	100	21	5	Positive
Thr	Thr	120	93.1	20	100	21	5	Positive
Thr	Thr	120	91.1	20	100	21	5	Positive
Thr	Thr	120	77.1	20	100	21	5	Positive
Trp	Trp	205	188	20	40	10	5	Positive
Trp	Trp	205	159	20	40	10	5	Positive
Trp	Trp	205	146	20	40	10	5	Positive
Trp	Trp	205	131.9	20	40	10	5	Positive
Tyr	Tyr	182	164.9	20	40	11	5	Positive
Tyr	Tyr	182	147	20	40	11	5	Positive
Tyr	Tyr	182	136	20	40	11	5	Positive
Tyr	Tyr	182	123	20	40	11	5	Positive
Val	Valin	118.2	72.2	20	60	10	5	Positive
Val	Valin	118.2	55.1	20	60	5	5	Positive

Table S4. HPLC – MS/MS conditions for separation and characterization of the chlorogenic acids (CQAs) and caffeine

Sample and Standards
10 mg samples were extracted with 1 ml 80% MeOH/ 20% distilled water containing 1 % formic acid for 30 min at room temperature under shaking. The samples were at centrifuged for 10 min at 9300 x g (Sigma, Saint Louis, USA). The supernatant was diluted 1:10 with 80% MeOH/ 20% distilled water containing 1 % formic acid for the extracts from the coffee beans. 5 µl were injected.

The calibration standards were: Chlorogenic acid and Caffeine (5-CQA, Sigma-Aldrich, Steinheim, Germany). Calibration was performed between – 1. 5-CQA: 5-40 µg/ml; 2. Caffeine 5-30 µg/ml.

HPLC Conditions:
Column: Prontosil 120-3-C18 ACE-EPS, 3 µm, 120 A, 250 x 3 mm (Bischoff Analytiktechnik und -geräte GmbH, Leonberg, Germany); Pressure limits 400 bar. Column temperature: 40°C
Eluents: A = 1 % Formic acid; B = Acetonitrile (Merck, Darmstadt, Germany); Flow rate: 0.5 ml/min;
Gradient: 0 min – 90 % A, 0-20 min; 90% A, 20-26 min; 90-70% A, 26-37 min, 70-64 %A; 37-38 min, 25% A; 38-41 min, 25% A; Post run 5 min (Total time. 46 min).
Sampler: 5 µl
Sampler Temperature: 10 °C
HPLC Sytem: Agilent Infinity 1260 System (binary pump, multicolumn thermostat, vial sampler VL, UV-Vis Dual Wavelength Detector (set at 280/325 nm)
Mass spectrometer: Agilent G6470A Series Triple Quad LC/MS (Agilent Technologies Sales & Services GmbH & Co.KG, Waldbronn, Germany)

MS/MS Conditions:
Collision Gas: Nitrogen
Pressure: 3.41E-5 Torr
Detection: MRM
Fragmentor Voltage: 80 V
Collision Energy (CE, eV) was individually optimized:
Cell Accelator Voltage: 5 kV
Dwell time: 20 ms

ESI Conditions:
Negative Ion-Mode
Temp. sheath gas: 275°C
Sheath Gas: Nitrogen
Flow-Rate (Gas): 11.0 l/min
Capillary (V): 4000 (+Ve); 3500 (-Ve)
Nebulizer pressure: 35.0 psi
For UV/Vis (280 nm / 325 nm) and MS/MS transitions: PTO

Table S4a: MRM Transition list for CQAs and caffeine

Compound Name	Precursor Ion	Charge	Product Ion	Charge	Dwell	Fragmen-tor	Collision Energy	Cell Accelerator Voltage	Polarity
Caffeine	195	M+	138	M+	20	80	20	5	Positive
Caffeine	195	M+	110,1	M+	20	80	25	5	Positive
Caffeine	195	M+	83,1	M+	20	80	35	5	Positive
CQA	353,1	M-	191,2	M-	20	80	20	5	Negative
CQA	353,1	M-	179	M-	20	80	20	5	Negative
CQA	353,1	M-	173	M-	20	80	20	5	Negative
CQA	353,1	M-	161,1	M-	20	80	20	5	Negative
CQA	353,1	M-	134,6	M-	20	80	20	5	Negative
CQA	353,1	M-	127,1	M-	20	80	20	5	Negative
Cys-CQA	476	M+	355	M+	20	80	10	5	Positive
Cys-CQA	476	M+	337	M+	20	80	10	5	Positive
Cys-CQA	476	M+	163	M+	20	80	10	5	Positive
Cys-CQA	476	M+	122	M+	20	80	10	5	Positive
Di-CQA	515	M-	353	M-	20	80	15	5	Negative
Di-CQA	515	M-	202,9	M-	20	80	35	5	Negative
Di-CQA	515	M-	191	M-	20	80	40	5	Negative
Di-CQA	515	M-	178,9	M-	20	80	40	5	Negative
Di-CQA	515	M-	172,8	M-	20	80	40	5	Negative
Di-CQA	515	M-	160,9	M-	20	80	35	5	Negative
Di-CQA	515	M-	135	M-	20	80	35	5	Negative
F-CQA	529,2	M-	367,2	M-	20	80	15	5	Negative
F-CQA	529,2	M-	353,3	M-	20	80	15	5	Negative
F-CQA	529,2	M-	335	M-	20	80	25	5	Negative
F-CQA	529,2	M-	193	M-	20	80	35	5	Negative
F-CQA	529,2	M-	191	M-	20	80	25	5	Negative
F-CQA	529,2	M-	179	M-	20	80	35	5	Negative
F-CQA	529,2	M-	173,1	M-	20	80	40	5	Negative
FQA	367	M-	193	M-	20	80	20	5	Negative
FQA	367	M-	191	M-	20	80	15	5	Negative
FQA	367	M-	185	M-	20	80	20	5	Negative
FQA	367	M-	173	M-	20	80	15	5	Negative
FQA	367	M-	137	M-	20	80	20	5	Negative
FQA	367	M-	133,7	M-	20	80	20	5	Negative
p-CoQA	337,5	M-	191	M-	20	80	15	5	Negative
p-CoQA	337,5	M-	172,8	M-	20	80	20	5	Negative
p-CoQA	337,5	M-	119,1	M-	20	80	35	5	Negative

Table S5. HPLC – MS/MS conditions for separation and characterization of the cysteine-chlorogenic acid-adducts (Cys-CQA)

Workflow

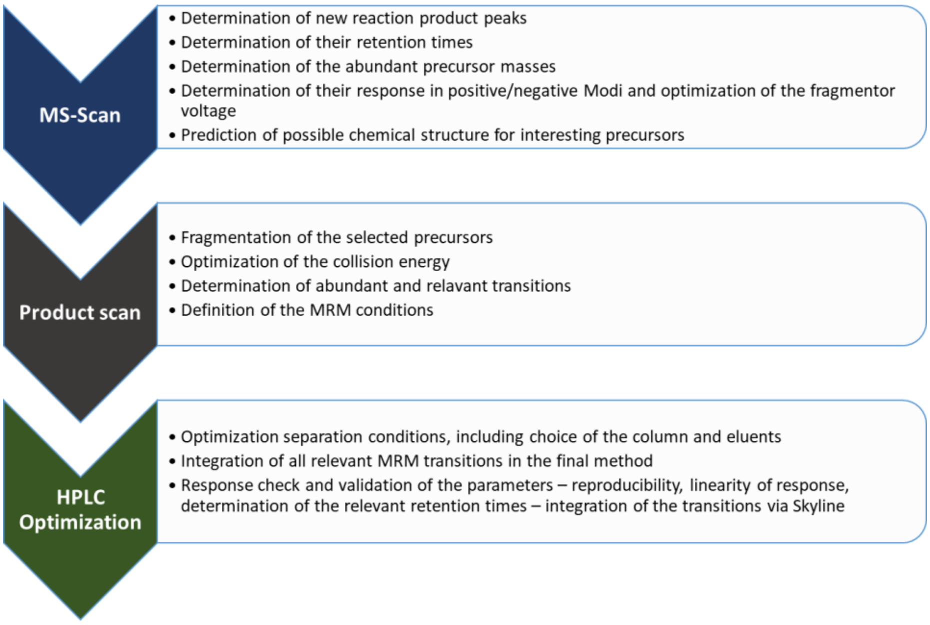

HPLC Conditions:

Column: Kinetex C8, 2,6 µm, 100 A, 150 x 4.60 mm (Phenomenex, Torrance, CA, USA); Pressure limits 400 bar; 0.5 ml/min at 90 % A = 248 bar; Column temperature: 40°C

Eluents: A = 0.1 % Formic acid; B = Methanol (Merck KGaA - Supelco, Darmstadt, Germany); Flow rate: 0.5 ml/min;

Gradient: 0 min – 100 % A, 0-5 min; 100% A, 5-20 min; 100-50% A, 20-21 min, 50-5% A; 21-24 min, 5% A; 24-25 min, 5-100 % A; 25-28 min, 100% A, Post run 6 min (Total time. 34 min).

Sampler: 1-10 µl

Sampler Temperature: 10 °C

HPLC Sytem: Agilent Infinity 1260 System (binary pump, multicolumn thermostat, vial sampler VL, UV-Vis Dual Wavelength Detector (set at 280/325 nm)

Mass spectrometer: Agilent G6470A Series Triple Quad LC/MS (Agilent Technologies Sales & Services GmbH & Co.KG, Waldbronn, Germany)

MS/MS Conditions:

Collision Gas: Nitrogen

Pressure: 3.41E-5 Torr

Detection: MRM

Fragmentor Voltage: 80 V

Collision Energy (CE, eV) was individually optimized:

Cell Accelator Voltage: 5 kV

Dwell time: 20 ms

ESI Conditions:
Negative/Positive Ion-Mode
Temp. sheath gas: 275°C
Sheath Gas: Nitrogen
Flow-Rate (Gas): 11.0 l/min
Capillary (V): 4000 (+Ve); 3500 (-Ve)
Nebulizer pressure: 35.0 psi

Table S5a. MRM Transition list for Cys-CQA adducts

Compound Name	Precursor Ion	Product Ion	Dwell	Fragmentor	Collision Energy	Cell Accelerator Voltage	Polarity
CQA	353,1	179	20	80	20	5	Negative
CQA	353,1	173	20	80	20	5	Negative
CQA	353,1	161,1	20	80	20	5	Negative
CQA	353,1	134,6	20	80	20	5	Negative
CQA	353,1	127,1	20	80	20	5	Negative
CQA-Dimer	707	353	20	120	20	5	Negative
CQA-Dimer	707	191	20	120	40	5	Negative
CQA-Dimer	707	179	20	120	40	5	Negative
CQA-Dimer	707	173	20	120	40	5	Negative
Cys-CQA	476	355	20	80	10	5	Positive
Cys-CQA	476	337	20	80	10	5	Positive
Cys-CQA	476	163	20	80	10	5	Positive
Cys-CQA	476	122	20	80	10	5	Positive
Cys-CQA	474	353	20	80	10	5	Negative
Cys-CQA-Dimer	951	476	20	120	15	5	Positive
Cys-CQA-Dimer	951	355	20	120	30	5	Positive
Cys-CQA-Dimer	951	337,1	20	120	40	5	Positive
Cys-CQA-Dimer	951	216,8	20	120	40	5	Positive
Cys-CQA-Dimer	951	163	20	120	40	5	Positive
Cys-CQA-Dimer	951	136,8	20	120	40	5	Positive
Cys-CQA-Dimer	951	121,9	20	120	40	5	Positive
CYSTEIN	122	105	20	80	10	5	Positive
CYSTEIN	122	95,9	20	135	10	5	Positive
CYSTEIN	122	87	20	80	20	5	Positive
CYSTEIN	122	82	20	135	15	5	Positive
CYSTEIN	122	76	20	80	20	5	Positive
CYSTEIN	122	56	20	135	15	5	Positive
CYSTIN	241	152	20	135	10	5	Positive
CYSTIN	241	122	20	135	15	5	Positive
CYSTIN	241	120	20	135	15	5	Positive
CYSTIN	241	74	20	135	25	5	Positive

Table S6. HPLC – MS/MS conditions for separation and characterization of the N-acetyl-cysteine-chlorogenic acid-adducts (NAC-CQA)

HPLC Conditions:

Column: Kinetex C8, 2,6 µm, 100 A, 150 x 4.60 mm (Phenomenex, Torrance, CA, USA); Pressure limits 400 bar; 0.5 ml/min at 90 % A = 248 bar; Column temperature: 40°C

Eluents: A = 0.1 % Formic acid; B = Methanol (Merck KGaA - Supelco, Darmstadt, Germany); Flow rate: 0.5 ml/min;

Gradient: 0 min – 85 % A, 0-5 min; 85% A, 5-20 min; 85-50% A, 20-21 min, 50-5% A; 21-24 min, 5% A; 24-25 min, 5-85 % A; 25-28 min, 85% A, Post run 6 min (Total time. 34 min).

Sampler: 1-10 µl

Sampler Temperature: 10 °C

HPLC Sytem: Agilent Infinity 1260 System (binary pump, multicolumn thermostat, vial sampler VL, UV-Vis Dual Wavelength Detector (set at 280/325 nm)

Mass spectrometer: Agilent G6470A Series Triple Quad LC/MS (Agilent Technologies Sales & Services GmbH & Co.KG, Waldbronn, Germany)

MS/MS Conditions:

Collision Gas: Nitrogen

Pressure: 3.41E-5 Torr

Detection: MRM

Fragmentor Voltage: 80 V

Collision Energy (CE, eV) was individually optimized:

Cell Accelator Voltage: 5 kV

Dwell time: 20 ms

ESI Conditions:

Positibe7Negative Ion-Mode

Temp. sheath gas: 275°C

Sheath Gas: Nitrogen

Flow-Rate (Gas): 11.0 l/min

Capillary (V): 4000 (+Ve); 3500 (-Ve)

Nebulizer pressure: 35.0 psi

Table S6a. MRM Transition list for NAC-CQA adducts

Compound Name	Precursor Ion	Product Ion	Dwell	Fragmentor	Collision Energy	Cell Accelerator Voltage	Polarity
CQA	353,1	179	20	80	20	5	Negative
CQA	353,1	173	20	80	20	5	Negative
CQA	353,1	161,1	20	80	20	5	Negative
CQA	353,1	134,6	20	80	20	5	Negative
CQA	353,1	127,1	20	80	20	5	Negative
CQA-Dimer	707	353	20	120	20	5	Negative
CQA-Dimer	707	191	20	120	40	5	Negative
CQA-Dimer	707	179	20	120	40	5	Negative
CQA-Dimer	707	173	20	120	40	5	Negative
Di-NAC	323	194	20	80	10	5	Negative
Di-NAC	323	162	20	80	10	5	Negative
Di-NAC	323	128	20	80	10	5	Negative
Di-NAC	323	116	20	80	10	5	Negative
Di-NAC-CQA-1033	1033	516	20	80	20	5	Negative
Di-NAC-CQA-1033	1033	353	20	80	40	5	Negative
Di-NAC-CQA-1033	1033	191	20	80	40	5	Negative
Di-NAC-CQA-1033	1033	178,6	20	80	40	5	Negative
NAC	164	146	20	80	10	5	Positive
NAC	164	122	20	80	10	5	Positive
NAC	164	118	20	80	10	5	Positive
NAC	164	105	20	80	10	5	Positive
NAC-CQA-514	514	385	20	80	20	5	Negative
NAC-CQA-514	514	192,8	20	80	20	5	Negative
NAC-CQA-514	514	191	20	80	40	5	Negative
NAC-CQA-514	514	167	20	80	40	5	Negative
NAC-CQA-516	516	353	20	80	10	5	Negative
NAC-CQA-516	516	191	20	80	40	5	Negative
NAC-CQA-516	516	179	20	80	40	5	Negative
NAC-CQA-516	516	135	20	80	40	5	Negative

Table S7. HPLC – MS/MS conditions for separation and characterization of the glutathione-chlorogenic acid-adducts (GSH-CQA)

HPLC Conditions:

Column: Kinetex C8, 2,6 µm, 100 A, 150 x 4.60 mm (Phenomenex, Torrance, CA, USA); Pressure limits 400 bar; Column temperature: 30°C; Säule Nr. 40.

Eluents: A = 0.1 % Formic acid; B = Methanol (Merck KGaA - Supelco, Darmstadt , Germany); Flow rate: 0.5 ml/min;

Gradient: 0 min – 100 % A, 0-5 min; 100% A, 5-15 min; 100-80% A, 15-16 min, 80-50% A; 16-21 min, 50-5% A; 21-24 min, 5% A; 24-25 min, 5-100 % A; 25-28 min, 100% A, Post run 4 min (Total time. 32 min).

Sampler: 1 µl

Sampler Temperature: 10 °C

HPLC Sytem: Agilent Infinity 1260 System (binary pump, multicolumn thermostat, vial sampler VL, UV-Vis Dual Wavelength Detector (set at 280/325 nm)

Mass spectrometer: Agilent G6470A Series Triple Quad LC/MS (Agilent Technologies Sales & Services GmbH & Co.KG, Waldbronn, Germany)

MS/MS Conditions:

Collision Gas: Nitrogen

Pressure: 3.41E-5 Torr

Detection: MRM

Fragmentor Voltage: 80 V

Collision Energy (CE, eV) was individually optimized:

Cell Accelator Voltage: 5 kV

Dwell time: 20 ms

ESI Conditions:

Positive/Negative Ion-Mode

Temp. sheath gas: 275°C

Sheath Gas: Nitrogen

Flow-Rate (Gas): 11.0 l/min

Capillary (V): 4000 (+Ve); 3500 (-Ve)

Nebulizer pressure: 35.0 psi

Table S7a. MRM Transition list for GSH-CQA adducts

Compound Name	Precursor Ion	Product Ion	Dwell	Fragmentor	Collision Energy	Cell Accelerator Voltage	Polarity
CQA	353,1	179	20	120	20	5	Negative
CQA	353,1	173	20	120	20	5	Negative
CQA	353,1	161,1	20	120	20	5	Negative
CQA	353,1	134,6	20	120	20	5	Negative
CQA	353,1	127,1	20	120	20	5	Negative
CQA-Dimer	707	353	20	120	20	5	Negative
CQA-Dimer	707	191	20	120	40	5	Negative
CQA-Dimer	707	179	20	120	40	5	Negative
CQA-Dimer	707	173	20	120	40	5	Negative
GSH	308,33	233,1	20	120	10	5	Positive
GSH	308,33	179,1	20	120	10	5	Positive
GSH	308,33	162	20	120	20	5	Positive
GSH	308,33	84,1	20	120	30	5	Positive
GSH	308,33	76,1	20	120	30	5	Positive
GSH-CQA	662	355	200	100	20	5	Positive
GSH-CQA	662	308	200	100	25	5	Positive
GSH-CQA	662	233	200	100	25	5	Positive
GSH-CQA	662	179	200	100	25	5	Positive
GSH-CQA	662	163	200	100	25	5	Positive
GSH-CQA	660	353	200	100	20	5	Negative
GSSG	613,7	484,2	20	120	20	5	Positive
GSSG	613,7	355,2	20	120	20	5	Positive
GSSG	613,7	231,1	20	120	30	5	Positive
GSSG	613,7	177	20	120	30	5	Positive

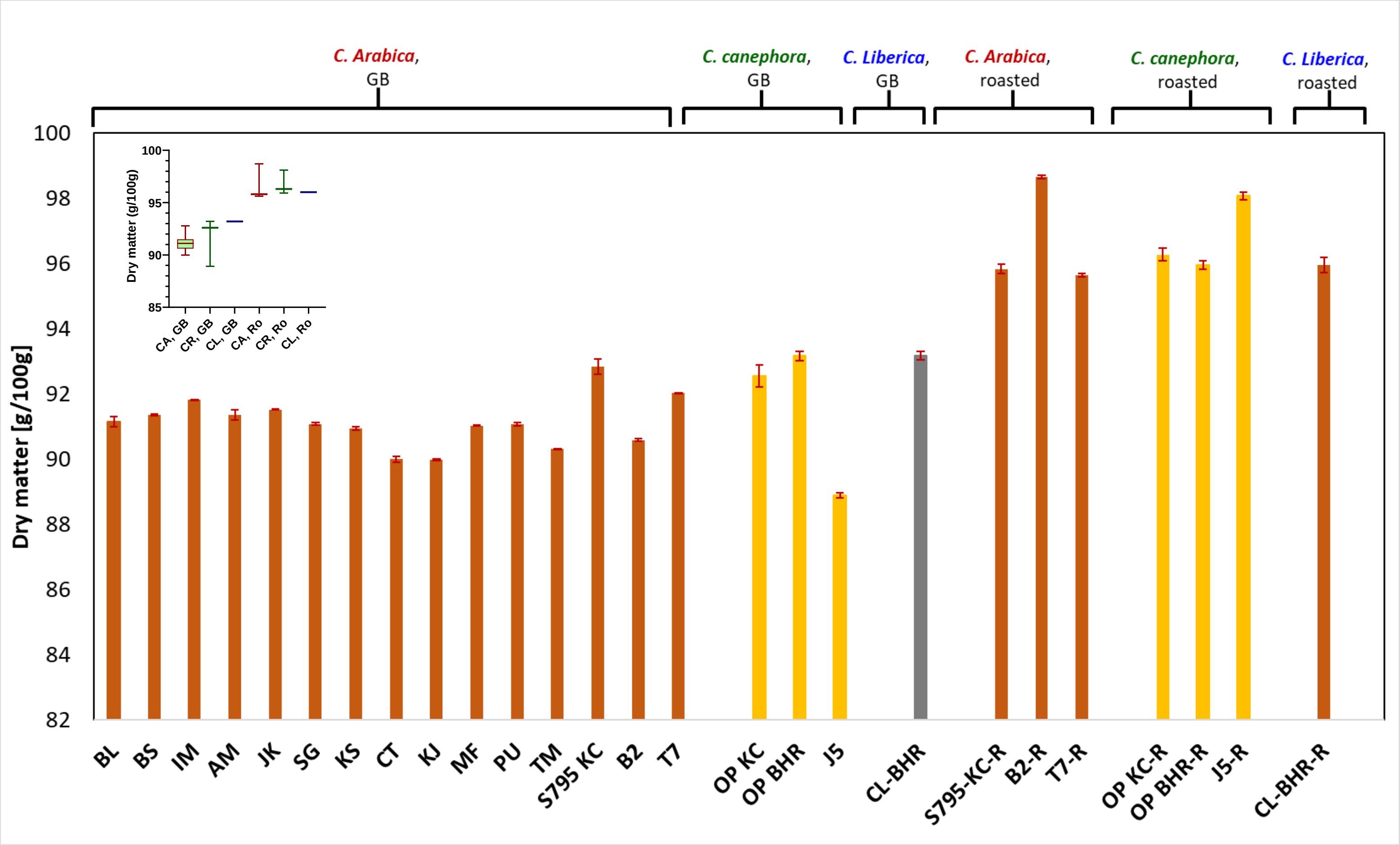

Figure S1 Dry matter of the coffee samples; abbreviations: BHR - Balehonnur; KC - Kerkiecoondah; OP - Old Paradenia, dry matter, 105°C. The Inset shows the range for the different samples. Abbreviations: CA = *C. Arabica*; CR = *C. canephora*; CL = *C. Liberica*; GB = Green Beans; Ro = Roasted. Sample code is given in Table S2.

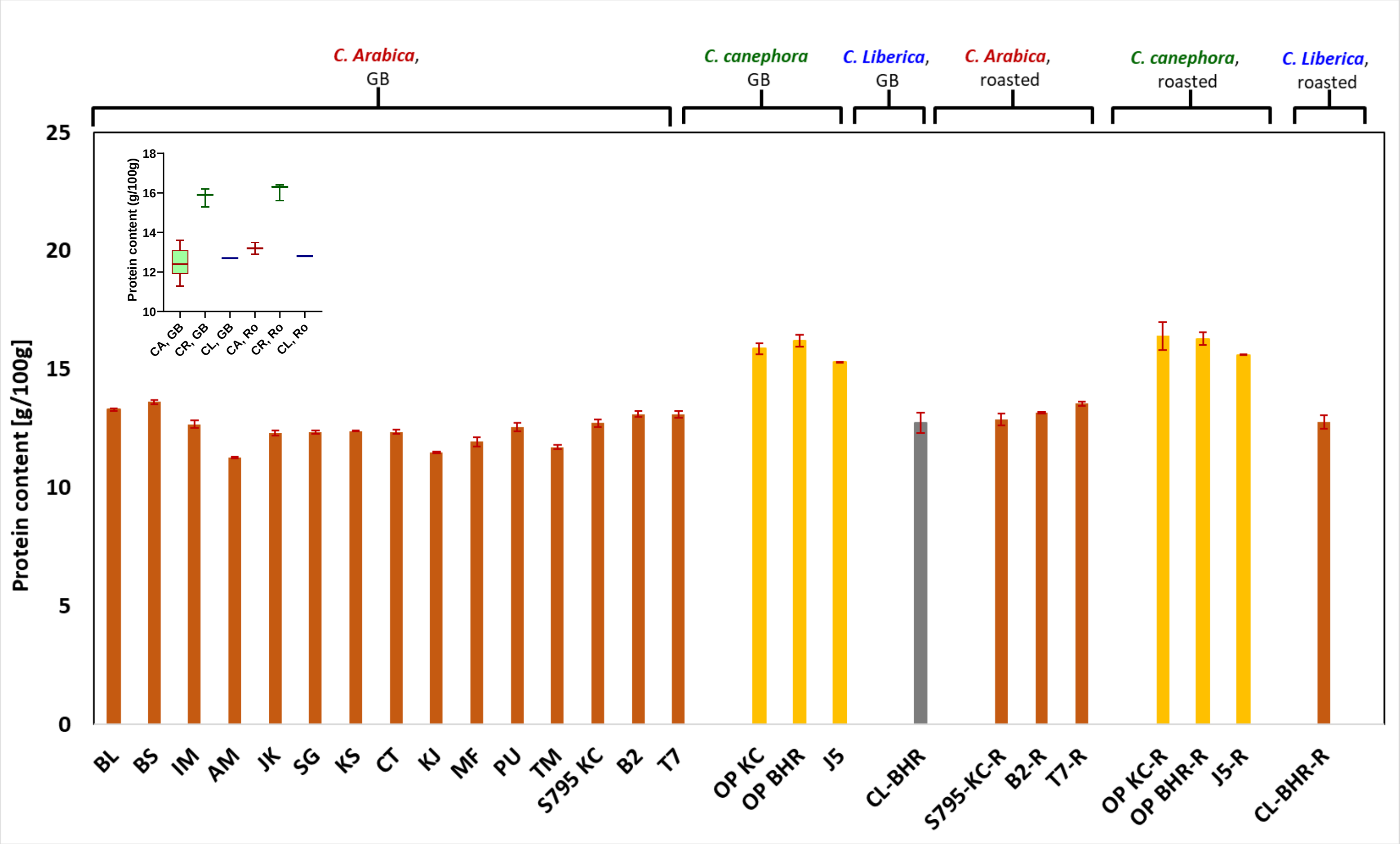

Figure S2 Protein content according to Kjeldahl in the coffee varieties; including caffeine (0.7 - 1.8 %), trigonelline (0.3 - 1.2 % [1]) and free amino acids (raw 0.07 - 0.11 %, roasted 0.009 - 0.01 %; 0.2 - 0.8 % [1]). The Inset shows the range for the different samples. Abbreviations: CA = C. Arabica; CR = C. canephora; CL = C. Liberica; GB = Green Beans; Ro = Roasted. Sample code is given in Table S2.

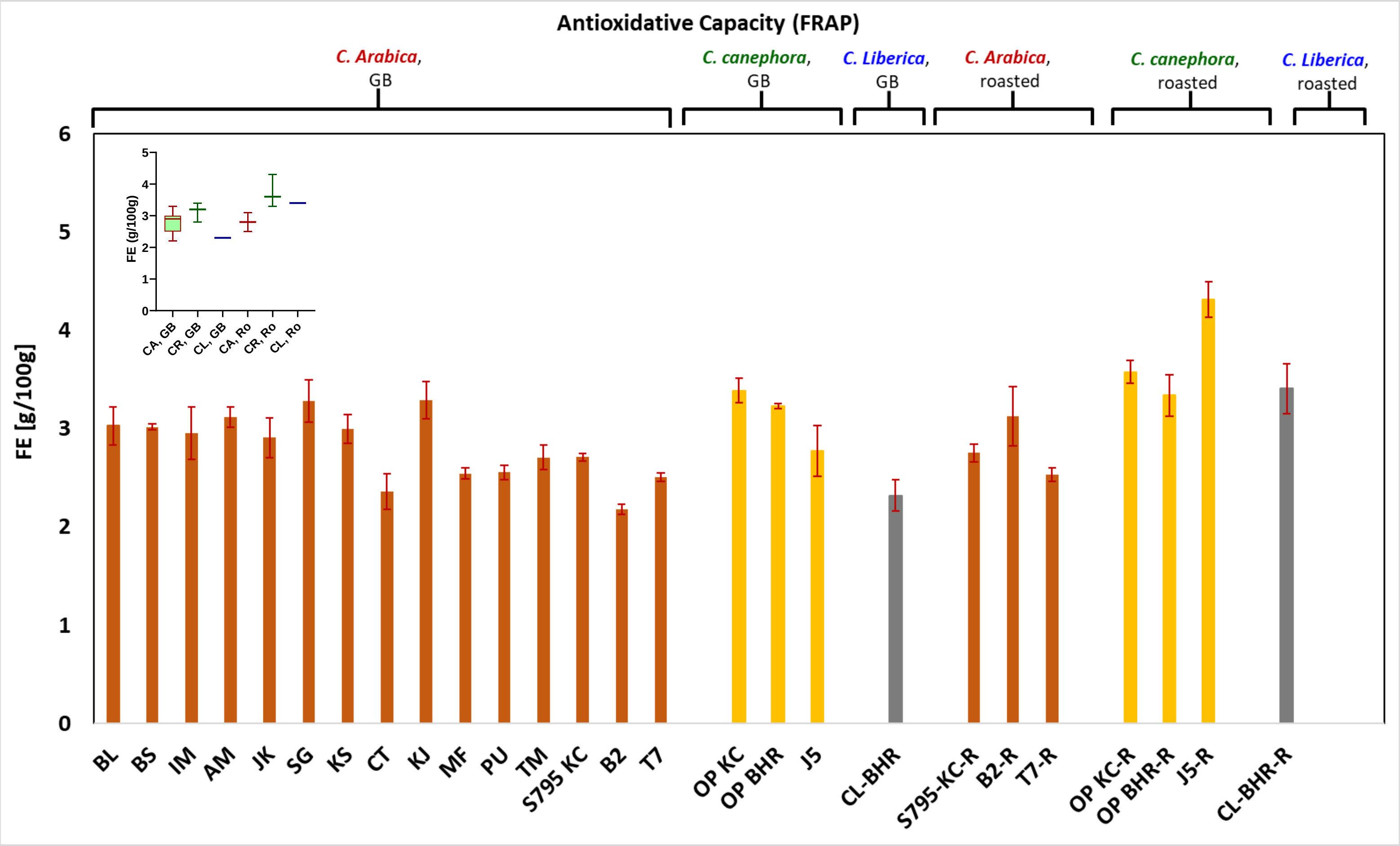

Figure S3 Antioxidant capacity of the coffee samples; abbreviations: FE - FRAP equivalent, FRAP - Ferric Reducing Ability of Plasma, GB - Green bean, OP - Old Paradenia. Sample code is given in Table S2.

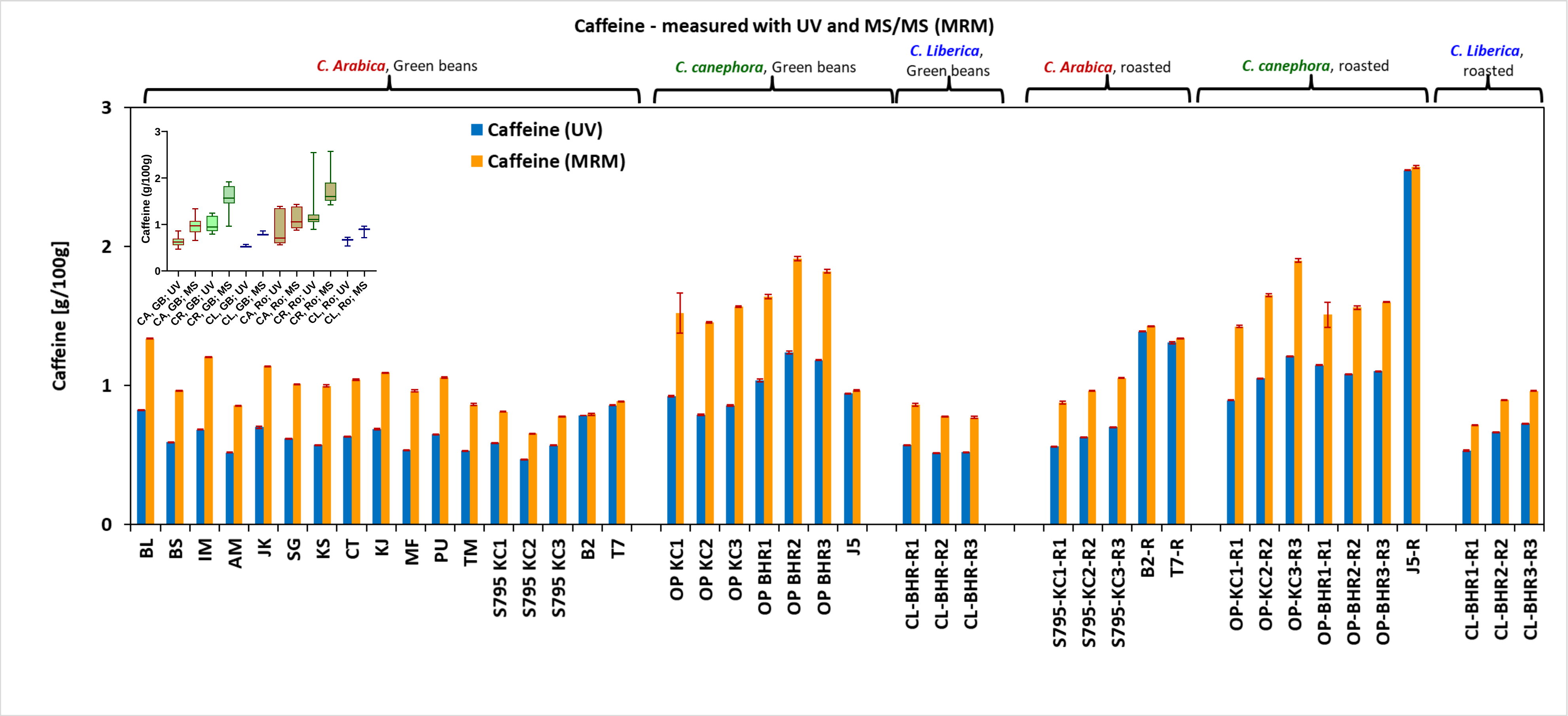

Figure S4 Caffeine content of the individual samples as measured by UV (280 nm) and MS/MS (MRM). The Inset shows the range for the different samples. Abbreviations: CA = *C. Arabica*; CR = *C. Robusta*; CL = *C. Liberica*; GB = Green Beans; Ro = Roasted; UV = detection at 280 nm; MS = detection with Mass Spectrometry. Sample code is given in Table S2.

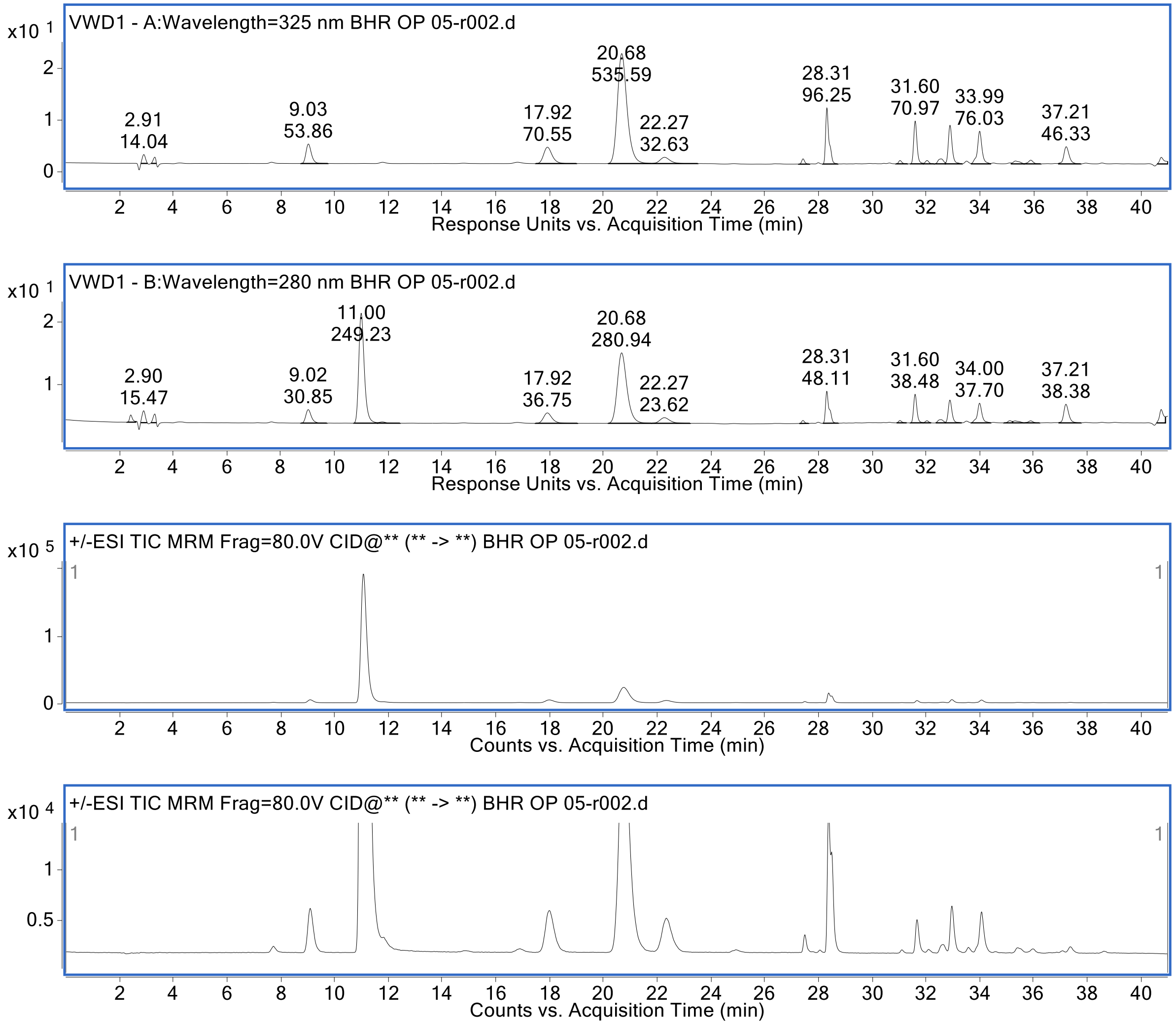

Figure S5 Chromatograms of the analyzed compounds, above (UV 325 nm), followed by UV measurement at 280 nm and finally the TIC (Total ion Chromatograms) from MS/MS Data. The last segment shows an enlargement of the third MS chromatogram (showing mainly the high response of caffeine at retention time of 11.2 min) from above it to illustrate the lower amounts of CQAs present as compared to caffeine.

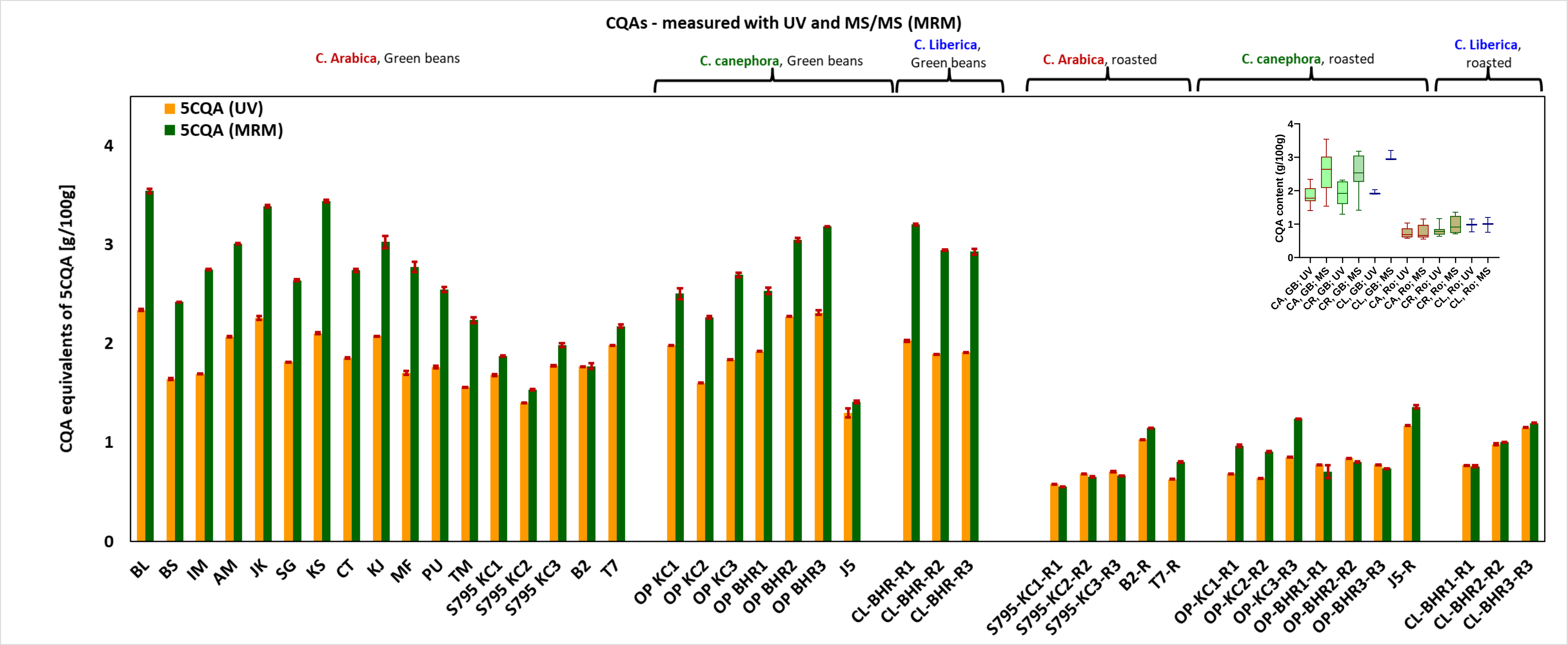

Figure S6 Chlorogenic acid content of the individual samples as measured by UV (325nm) and MS/MS (MRM). The Inset shows the range of 5 CQA for the different samples. Abbreviations: 5-CQA = 5-caffeoylquinic acid; CA = *C. Arabica*; CR = *C. Robusta*; CL = *C. Liberica*; GB = Green Beans; Ro = Roasted; UV = detection at 325 nm; MS = detection with Mass Spectrometry. Sample code is given in Table S2.

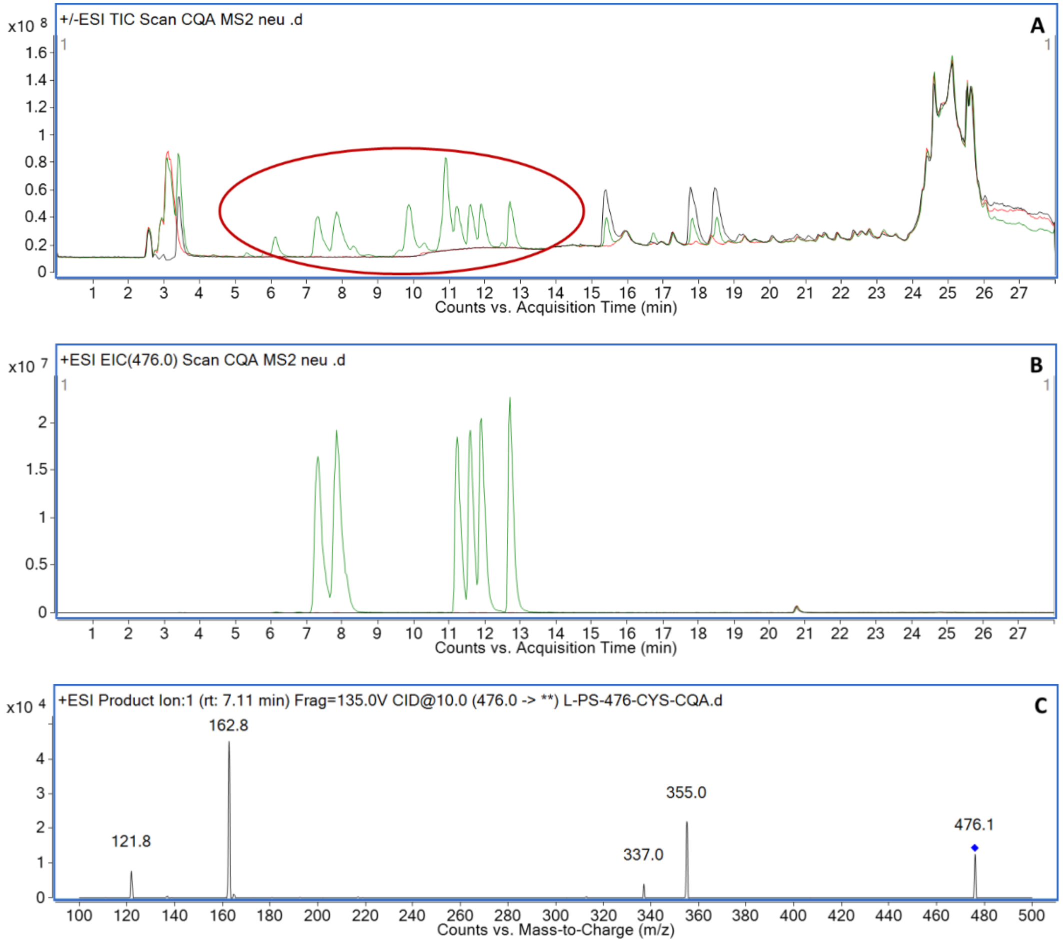

Figure S7 Identification of Cys-CQA adducts. A: Superimposed chromatograms of the MS scans of the three standard solutions with 30 mM chlorogenic acid, 100 mM cysteine or their 1:1 mixture; colors: green - cysteine + chlorogenic acid; black - chlorogenic acid; red – cysteine; B: Extracted ion Chromatogram for m/z = 476 showing the Cys-CQA adducts; C: Exemplary fragmentation pattern for Cys-CQA adducts.

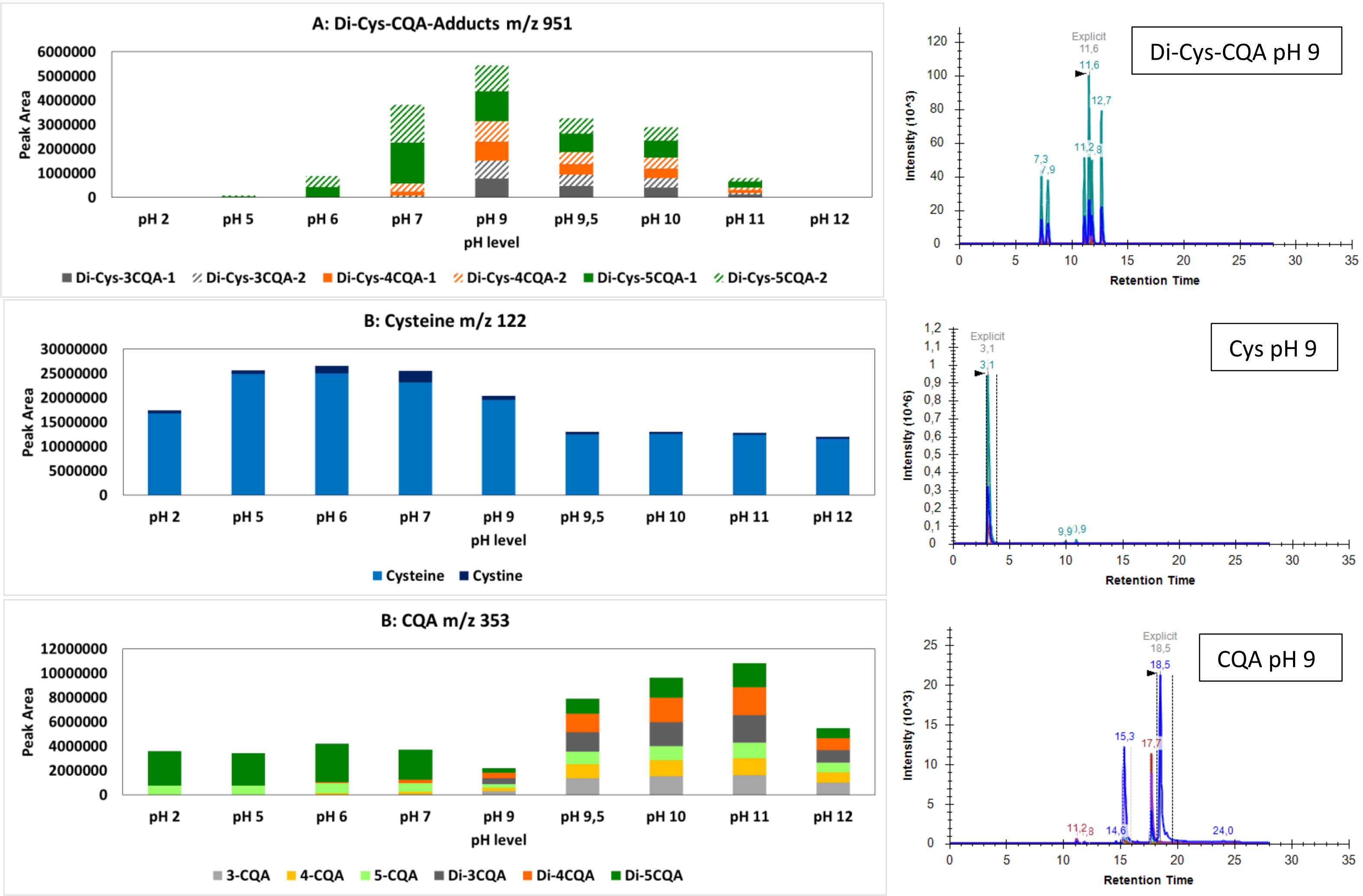

Figure S8 Chromatograms and amounts (peak area) of reaction products occurring during the incubation of CQA and Cys at different pH conditions with 30 mM chlorogenic acid and 100 mM cysteine in their 1:1 mixture. A: Formation of di-Cys-CQA adducts at different pH values; B: Cysteine distribution at different pH values; C: Chlorogenic acid distribution at different pH values; abbreviations: CQA - caffeoylquinic acid, Cys – Cyteine, Di-CQA - dimer of caffeoylquinic acid.

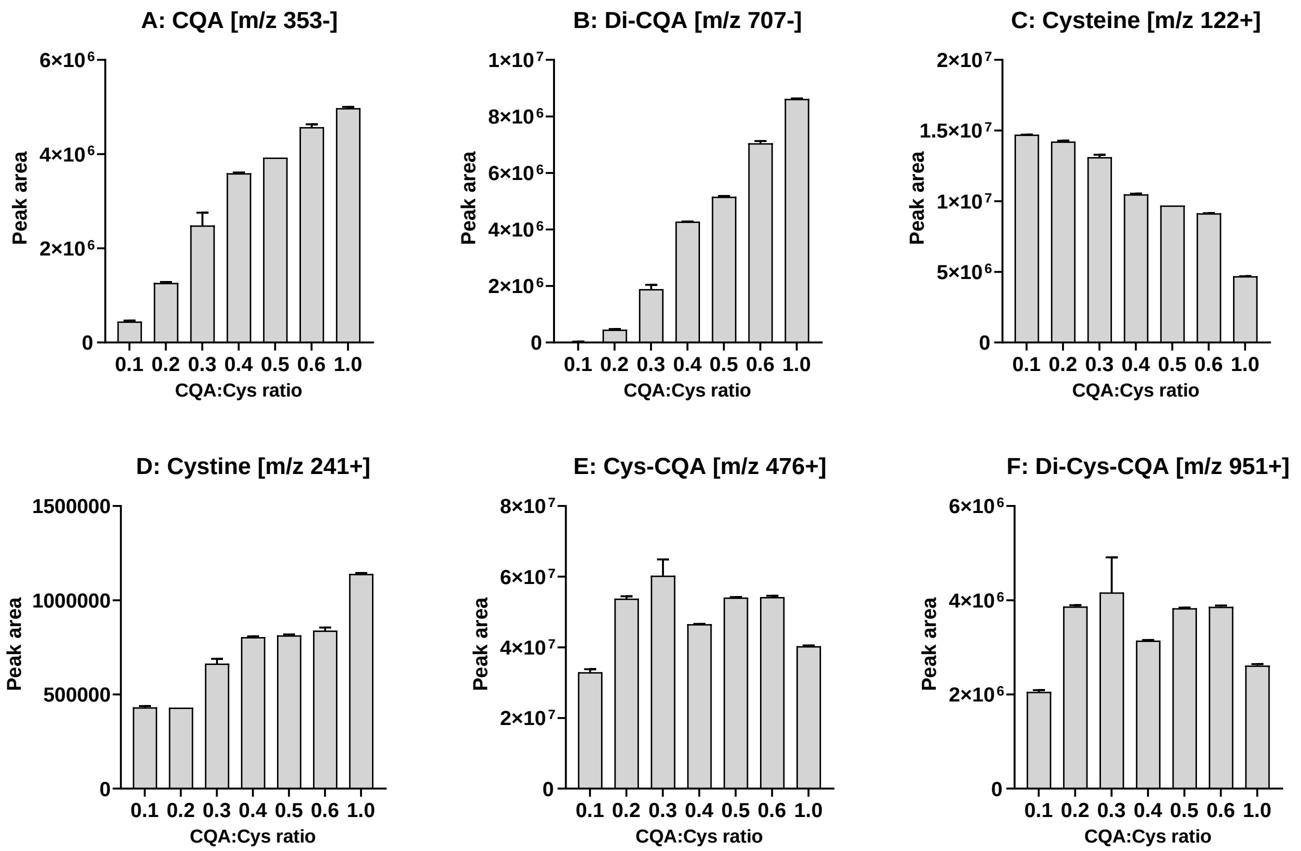

Figure S9 Concentration dependence of the adduct formation after incubation for 20h at different concentration ratios of the reactant molecules mixed 1:1 (v/v) at the optimum pH 9 and at room temperature. The final concentrations were reduced to half of those depicted in the figures after mixing. A: Change in CQA; B: Change in Di-CQA; C: Change in cysteine and D: in cystine; as well as the corresponding adduct formation E: at m/z 476 and F: at m/z 951; abbreviations: CQA - caffeoylquinic acid, Cys – Cyteine, Di-CQA - dimer of caffeoylquinic acid.

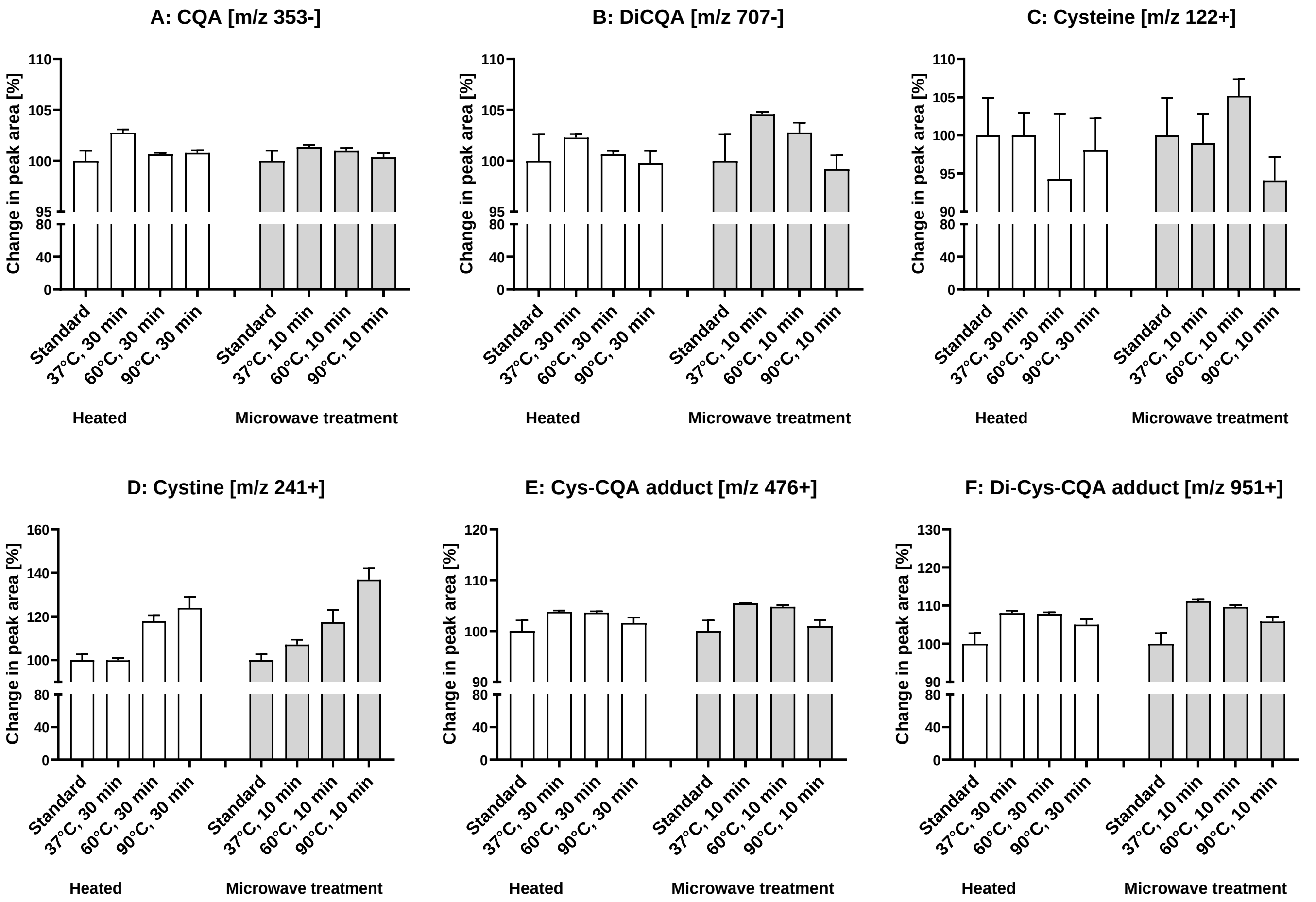

Figure S10 Stability of the adducts after incubation of 30 mM chlorogenic acid and 100 mM cysteine in their 1:1 mixture for 20h at the optimum pH 9 and at room temperature. The mixtures were acidified to stop their reaction. A: Change in CQA; B: Change in Di-CQA; C: Change in cysteine and D: in cystine; as well as the corresponding adduct formation E: at m/z 476 and F: at m/z 951; Values as mean ± standard deviation (* $p < 0.05$; ** $p < 0.05$; t-test); abbreviations: CQA - caffeoylquinic acid, Cys – Cyteine, Di-CQA - dimer of caffeoylquinic acid.

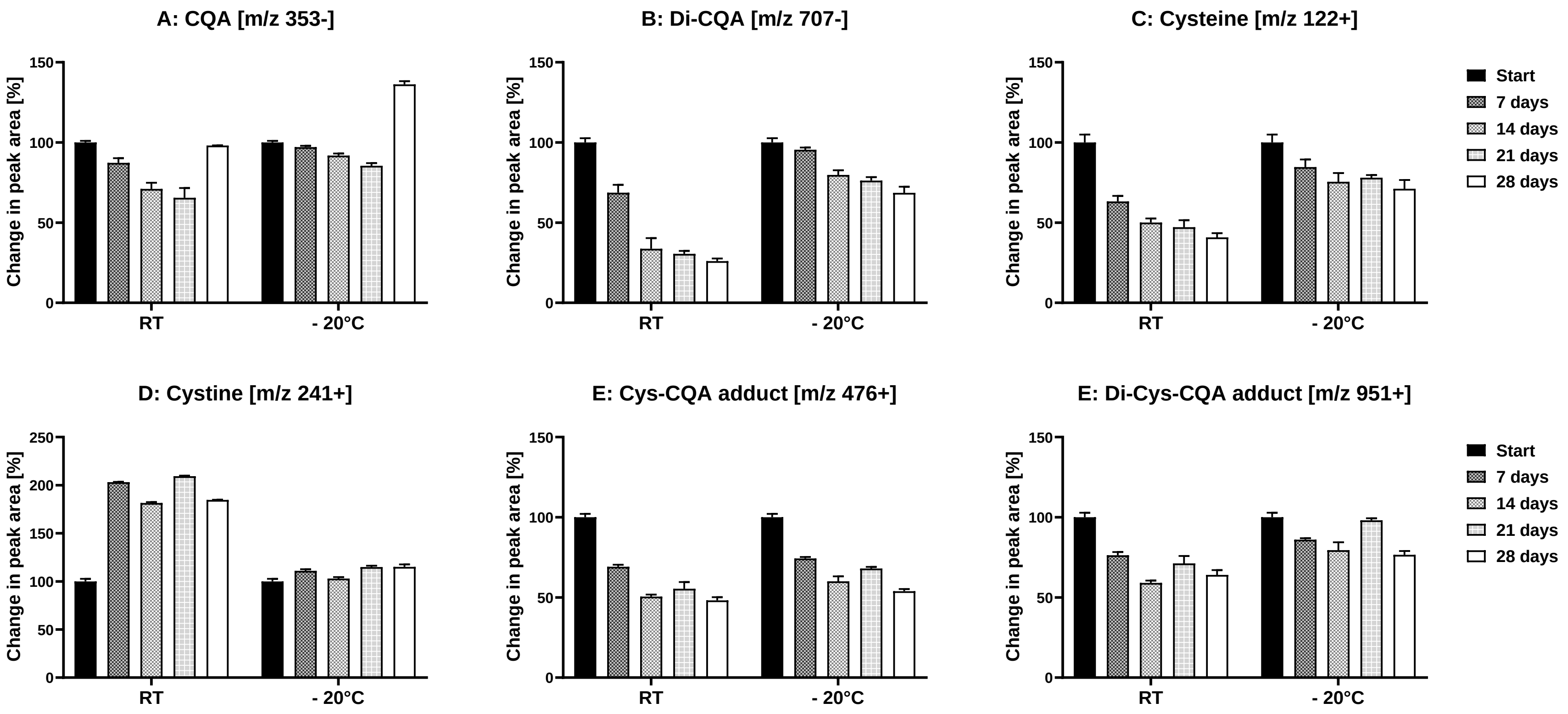

Figure S11 Long-term effect of storage of the adducts at room temperature (RT) and -20°C after incubation of 30 mM chlorogenic acid and 100 mM cysteine in their 1:1 mixture for 20h at the optimum pH 9 and at room temperature. The mixtures were acidified to stop their reaction. A: Change in CQA; B: Change in Di-CQA; C: Change in cysteine and D: in cystine; as well as the corresponding adduct formation E: at m/z 476 and F: at m/z 951; Values as mean ± standard deviation; abbreviations: CQA - caffeoylquinic acid, Cys – Cyteine, Di-CQA - dimer of caffeoylquinic acid.

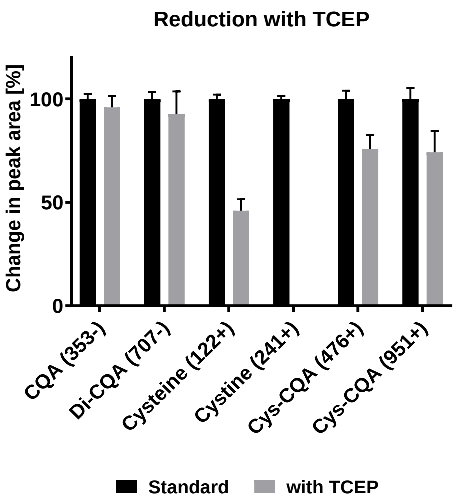

Figure S12 Effect of incubation with TCEP on the monitored compounds as a percentage change compared to samples to which no TCEP was added (Standard); abbreviations: CQA - caffeoylquinic acid, Cys – Cyteine, Di-CQA - dimer of caffeoylquinic acid, TCEP - Tris(2-carboxyethyl)phosphine hydrochloride.

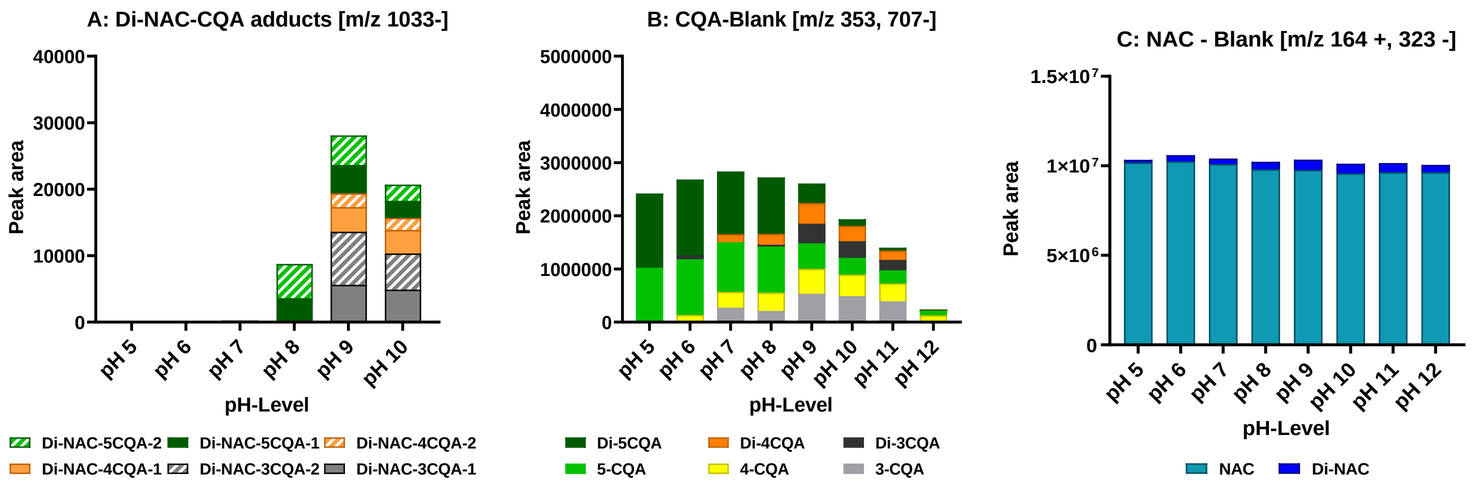

Figure S13 Amounts (peak area) of reaction products occurring during the incubation of CQA and NAC at different pH conditions with 30 mM chlorogenic acid and 100 mM NAC in their 1:1 mixture. A: Formation of di-NAC-CQA adducts at different pH values; B: NAC distribution at different pH values in the corresponding blanks; C: Chlorogenic acid distribution at different pH values in the corresponding blanks; abbreviations: CQA - caffeoylquinic acid, NAC - N-acetyl-cysteine, Di-CQA - dimer of caffeoylquinic acid.

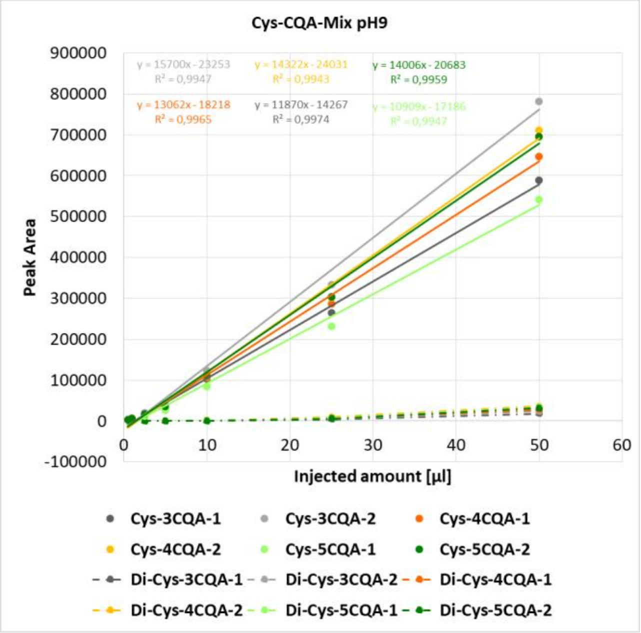

Figure S14 Linearity for the MS response for different amounts of the reaction products produced during the incubation of CQA and Cys at pH 9 with 30 mM chlorogenic acid and 100 mM Cys in their 1:1 mixture; abbreviations: CQA - caffeoylquinic acid, Cys - cysteine, Di-CQA - dimer of caffeoylquinic acid, MS - Mass spectrometry.

References

1. Belitz, H.-D.; Grosch, W.; Schieberle, P. *Food chemistry*. 6 ed.; springer: Berlin, Heidelberg, , 2008, 2009; p 969-1003.
